# Comparative microsomal proteomics of a model lung cancer cell line NCI-H23 reveals distinct differences between molecular profiles of 3D and 2D cultured cells

**DOI:** 10.18632/oncotarget.28072

**Published:** 2021-09-28

**Authors:** Jan A. Kaczmarczyk, Rhonda R. Roberts, Brian T. Luke, King C. Chan, Carly M. Van Wagoner, Robin A. Felder, Richard G. Saul, Colantonio Simona, Josip Blonder

**Affiliations:** ^1^Antibody Characterization Laboratory, Cancer Research Technology Program, Frederick National Laboratory for Cancer Research, Frederick, MD 21701, USA; ^2^Advanced Biomedical Computational Science, Frederick National Laboratory for Cancer Research, Frederick, MD 21701, USA; ^3^Protein Characterization Laboratory, Cancer Research Technology Program, Frederick National Laboratory for Cancer Research, Frederick, MD 21701, USA; ^4^Department of Pathology, University of Virginia School of Medicine, Charlottesville, VA 22908, USA; ^5^Current address: The Center for Cell Clearance, University of Virginia, Charlottesville, VA 22908, USA

**Keywords:** lung cancer, proteomics, 2D vs. 3D cultured cells, preclinical testing model, drug target discovery

## Abstract

Lung cancer is the leading cause of cancer-related deaths in the USA and worldwide. Yet, about 95% of new drug candidates validated in preclinical phase eventually fail in clinical trials. Such a high attrition rate is attributed mostly to the inability of conventional two-dimensionally (2D) cultured cancer cells to mimic native three-dimensional (3D) growth of malignant cells in human tumors.

To ascertain phenotypical differences between these two distinct culture conditions, we carried out a comparative proteomic analysis of a membrane fraction obtained from 3D- and 2D-cultured NSCLC model cell line NCI-H23. This analysis revealed a map of 1,166 (24%) protein species regulated in culture dependent manner, including differential regulation of a subset of cell surface-based CD molecules. We confirmed exclusive expression of CD99, CD146 and CD239 in 3D culture. Furthermore, label-free quantitation, targeting KRas proteoform-specific peptides, revealed upregulation of both wild type and monoallelic KRas4B^G12C^ mutant at the surface of 3D cultured cells.

In order to reduce the high attrition rate of new drug candidates, the results of this study strongly suggests exploiting base-line molecular profiling of a large number of patient-derived NSCLC cell lines grown in 2D and 3D culture, prior to actual drug candidate testing.

## INTRODUCTION

Lung carcinoma is the deadliest cancer in the United States and worldwide [1]. In 2019, the American Cancer Society reported 142,670 deaths from lung cancer in the United States. Lung cancer affects men and women equally, with an overall five-year survival rate of 19%. Histologically, 80–85% of all cases belong to non-small-cell lung cancer (NSCLC). The most common form of NSCLC is the adenocarcinoma of the lung [2]. Despite extensive research and economic investments, about 95% of new drugs against lung carcinoma eventually fail in clinical trials [3, 4]. While there are many hypotheses and explanations for this poor clinical translation rate, it is well accepted that conventionally used preclinical *in vitro* testing models (e.g., lung cancer cell lines) are incapable of reproducing the growth of malignant cells in human tumors *in vivo*. Hence, after the initial discovery phase, preclinical testing is heavily burdened by excessive proportion of false positive results that drive the high lung cancer drug attrition rate [5]. Adherent two-dimensional (2D) *in vitro* cultures of human cancer cell line are the mainstay preclinical testing model used in lung cancer drug development and discovery [6]. In solid tumors, however, cancer cells grow in a three-dimensional (3D) environment. This makes the 2D cultured cells unable to truly reproduce the natural proliferation, migration, and/or drug permeation taking place in their innate 3D environment [7]. To circumvent this shortcoming, the 3D cancer cell culture have been proposed as a more accurate/relevant preclinical testing models, not only biomechanically but also at the genome, proteome, and metabolome level [7–9]. Typically, preclinical assessment of drug effectiveness relies on functional cell assays, incidental imaging procedures, and histopathological examinations [10]. To better understand and standardize results obtained in the preclinical phase, it is of prime interest to better characterize and compare molecular phenotypes of lung cancer cells grown in 2D- and 3D-culture. Thus, there is an outstanding and unmet need for better characterization of preclinical lung cancer models to facilitate development of more effective predictive therapeutic biomarkers involved in molecular pathways driving lung cancer tumorigenesis [11]. This approach also creates a pathway towards the development of a preclinical atlas depicting the molecular profiles of cell lines grown in 2D vs. 3D, information that is sorely needed to accurately monitor drug development. Contemporary MS-based proteomics [12–16] represents a potent technology capable of revealing alterations in protein level expression/regulation and changes in post-translational modifications associated with preclinical drug evaluation [17, 18]. Furthermore, the differential molecular phenotype of a NSCLC cell line membrane proteome grown in 2D- vs. 3D-culture is still missing.

Herein we report results of differential shotgun membrane proteomic analysis of the microsomal fraction obtained from a NSCLC model cancer cell line grown in both 2D and 3D culture. Comparative proteomics that relies on the off-line strong cation exchange (SCX)-based fractionation of tryptic digests [19] and HR/AM LC-MS [20] was employed for in-depth mapping of the membrane proteome of the NCI-H23 cancer cell line. Next, spectral counting-based and label-free quantitative proteomics was utilized to elucidate differences in protein expression between cells grown in 2D and 3D culture.

## RESULTS

### Mapping microsomal proteome of 2D- and 3D-grown NCI-H23 cells using comparative proteomic analysis

**Figure 1:**
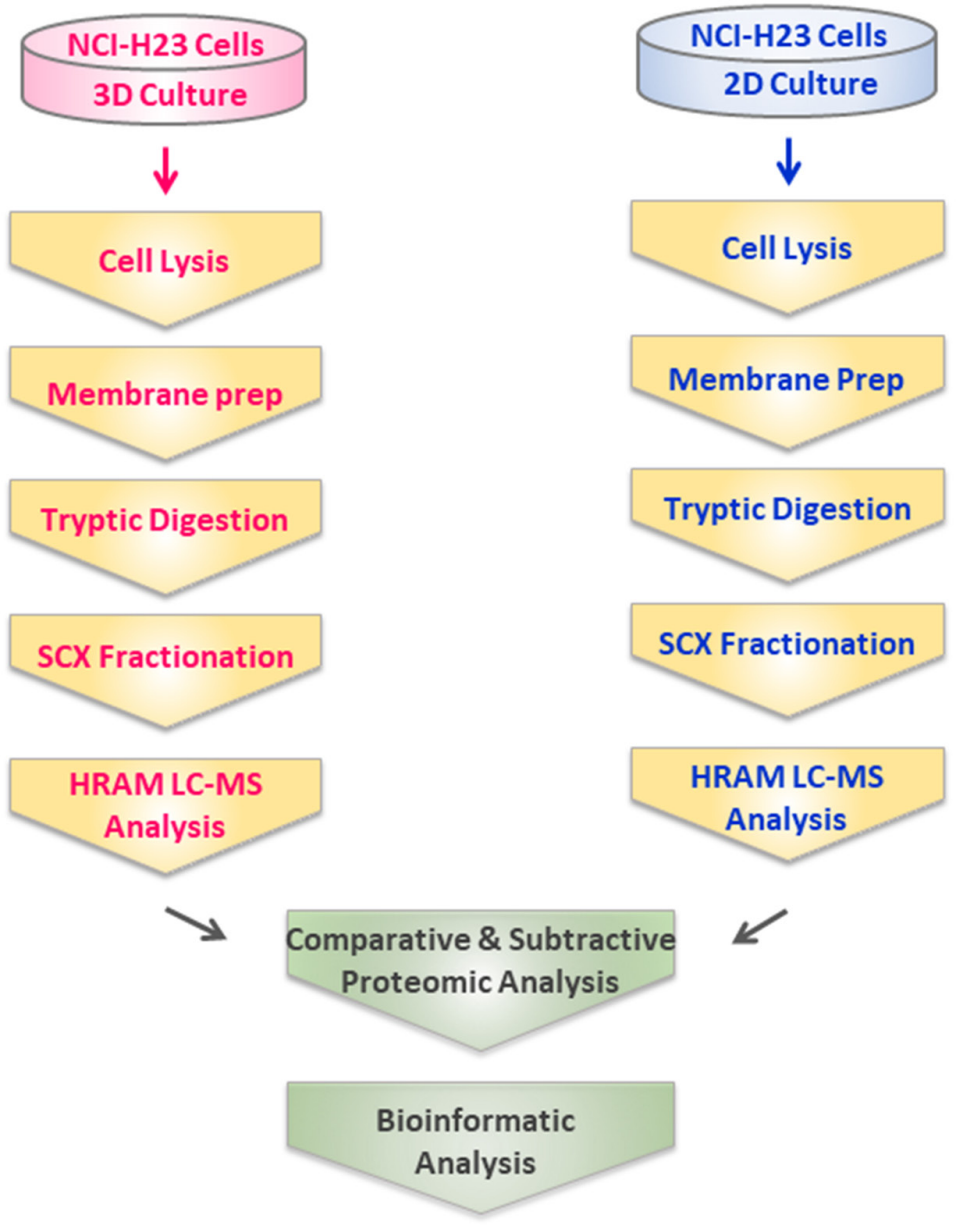
Experimental design and workflow for comparative profiling of 3D- and 2D-cultured NCI-H23 cells using comparative shotgun proteomics.

The aim of this investigation was to map differences in protein expression between microsomal fractions obtained from 2D- and 3D-grown NCI-H23 lung cancer model cell line bearing heterozygous KRas-G12C mutant https://www.cancer.gov/research/key-initiatives/ras/outreach/reference-reagents/cell-lines and elucidate biologically relevant alterations between these two cell culture conditions. Towards this goal, we used MS-based proteomics which has been established as an efficient approach for profiling of complex membrane protein mixtures [21–23]. The workflow is depicted in Figure 1. Briefly, tip sonication was used to disrupt intact cells and generate a microsomal fraction, typically comprising of double membrane-bound vesicles (e.g., plasma membrane, mitochondria, nuclei) and single membrane-bound vesicles (e.g., endoplasmic reticulum, lysosomes, Golgi apparatus). Finally, this crude microsomal fraction containing heterogenous membranous vesicles was isolated using ultracentrifugation [19]. Following tryptic digestion, the resulting peptide mixture was fractionated, and analyzed in duplicates using HR/AM LC-MS as previously described [19, 23]. The analysis resulted in the identification of a total of 4,180 and 4,444 protein groups depicted in Supplementary Tables 1–2, along with subcellular location annotations currently accessible in the Human Proteome atlas, from a total 30,986 and 35,360 tryptic peptides (Supplementary Tables 3–4) identified in microsomal fraction of NCI-H23 cells grown in 3D and 2D culture, respectively. A total of 1,345 (i.e., 32.2%) and 1,404 (i.e., 31.6%) were classified as integral membrane proteins in 3D- and 2D- cultured NCI-H23 using PSORT and TMHMM prediction algorithms (Supplementary Tables 5–6). The efficiency of the isolation protocol is exemplified by the enrichment and unambiguous identification of microsomal fraction-specific enzymes, NADPH-cytochrome P450 reductase (POR), lanosterol 14-alpha demethylase (CYP51A1) and cytochrome P450 2S1 (CYP2S1) found in microsomal fraction of both 3D and 2D cultured cells (Supplementary Tables 1–2). The search against the human CSPA found that 415 (i.e., 30.8%) and 434 (i.e., 30.9%) proteins identified in NCI-H23 cells grown in 3D and 2D culture, respectively, were cell surface proteins (Supplementary Tables 7–8), previously unambiguously identified by mass-spectrometry in more than 40 human cell types and catalogued in CSPA [24]. Of these, 53 (i.e., 12.7%) and 51 (i.e., 11.7%) were classified as cluster of designation (CD) molecules identified in NCI-H23 cells grown in 3D and 2D culture, respectively (Supplementary Tables 7–8).

### Classification of protein species identified in NCI-H23 cells grown in 3D and 2D culture

**Figure 2:**
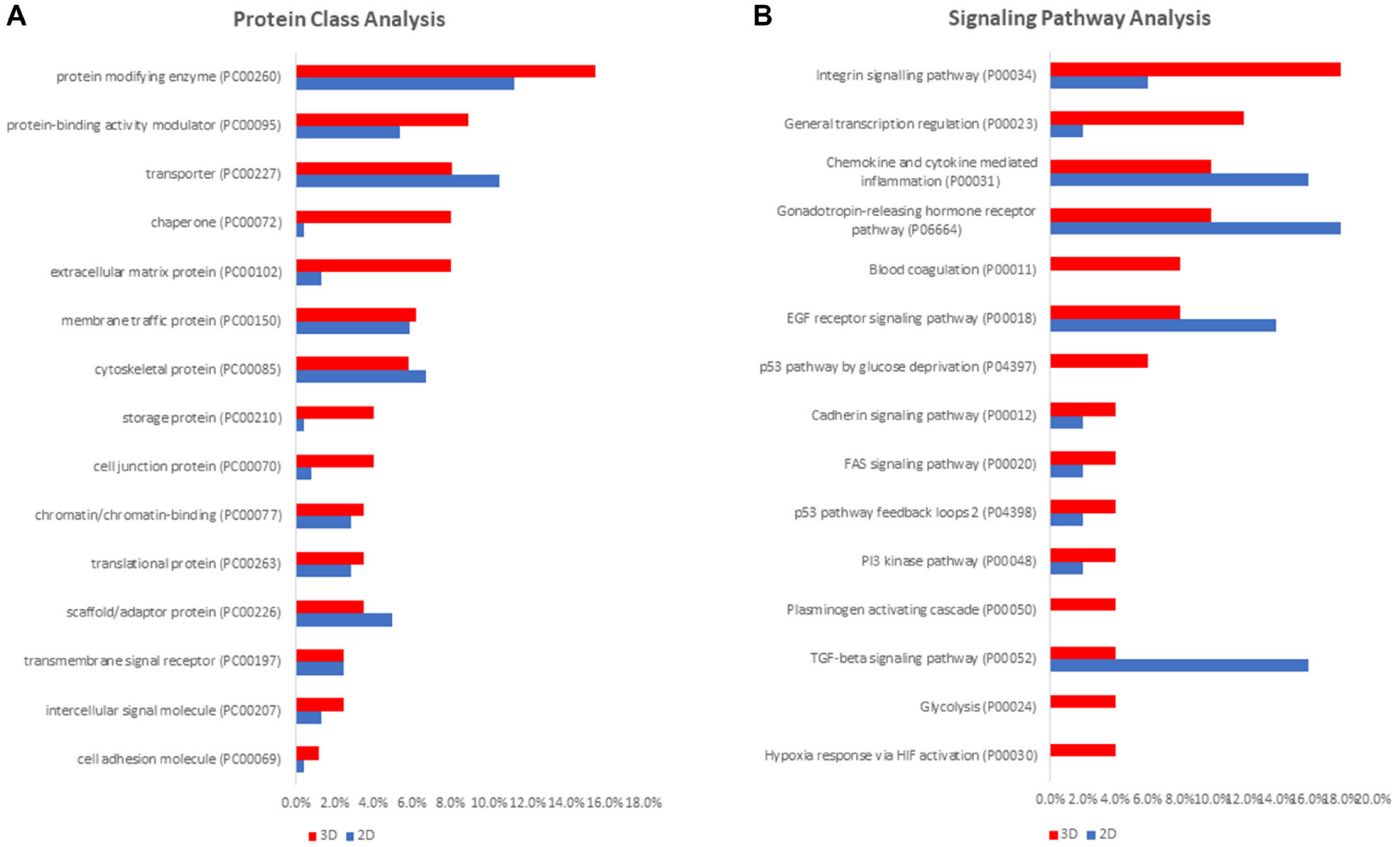
PANTHER bioinformatic analysis of differentially expressed proteins in 3D- and 2D-cultured NCI-H23 cells. Protein class analysis (**A**) and signaling pathway analysis (**B**).

We used PANTHER classification system to examine protein functions, protein classes and corresponding pathways in NCI-H23 cells grown in 3D and 2D culture. The PANTHER analysis of differentially expressed proteins exhibited similar distribution of molecular functions involving mainly catalytic activity, binding, molecular regulation, and transporter activity (Supplementary Figure 1A–1B). Significantly, the protein class analysis revealed enrichment of cell adhesion molecules, intercellular signaling molecules, cell junction proteins, and extracellular matrix proteins in 3D-grown cells. 2D-cultured cells showed a slight increase in the identification rate of transporters, cytoskeletal and scaffold proteins (Figure 2A). Comparative pathway analysis revealed significant activation of the integrin signaling pathway and of general transcription regulation pathway in 3D cultured cells, while TGF-beta signaling and EGF receptor signaling pathways were found increasingly activated in 2D-cultured cells (Figure 2B). Furthermore, p53 glucose deprivation pathway, glycolysis and hypoxia response via HIF activation were found activated exclusively in 3D cultured cells (Figure 2B). While the findings of the PANTHER molecular enrichment analysis are not highly specific, the enrichment of extracellular matrix proteins, cell junction proteins, intercellular signaling molecules and cell adhesion molecules are in agreement with the expected tissue-like phenotypical transition taking place in 3D culture [25]. Correspondingly, the unique enrichment of p53 glucose deprivation, hypoxia response via HIF activation and glycolysis pathways in the context of the 3D cell culture are in agreement with widespread hypoxia that is typical for 3D culture and solid tumors [26].

### Differential proteomic analysis exposes change in protein expression between 3D- and 2D-cultured cells

Comparative proteomic analysis revealed a subset of 3,846 protein species common to both cell culture conditions (Supplementary Figure 1C). Of these, a subset of 234 (i.e., 6.08%) protein species were found significantly dysregulated, marked red in Supplementary Table 9. Significantly, Ras proteoforms (i.e., KRas and NRas) were also found significantly dysregulated in culture-dependent manner (Supplementary Table 9). Subtractive proteomics (Supplementary Figure 1C) disclosed a subset of 334 (i.e., 7.9%) microsomal proteins identified exclusively in cells grown in 3D culture (Supplementary Table 10) and 598 (i.e., 13.4%) identified solely in 2-D cultured cells (Supplementary Table 11). Overall, this analysis revealed a subset of 1,166 (i.e., 24.4%) proteins regulated in a culture dependent manner (Supplementary Table 12) among non-redundantly identified protein species (i.e., 4,778) in both culture conditions (Supplementary Figure 1C). Furthermore, a total of 31 (i.e., 9.3%) and 50 (i.e., 8.7%) proteins identified solely in 3D (Supplementary Tables 13–14) and 2D grown cells respectively, were classified as genuine cell surface proteins by the CSPA.

Remarkably, in a subset of the top 10 most abundant cell surface proteins detected exclusively and/or found significantly upregulated in 3D cultured NCI-H23 cells depicted in Table 1, each protein detected exclusively in 3D cultured cells and the vast majority of proteins found significantly upregulated in 3D culture are known to reside in malignant tumor stroma (i.e., tumor microenvironment). On the other hand, most of the most abundant proteins found significantly upregulated and/or detected solely in 2D cultured cells (Table 2), were found to be expressed by malignant cells residing in tumor parenchyma (i.e., tumor proper). Intriguingly, a substantial number of these protein species are annotated as CD molecules and were found to be expressed in culture dependent manner. Of these, basal CD99 antigen, CD109 antigen, CD146 (i.e., cell surface glycoprotein, MCAM) and CD239 (i.e., basal cell adhesion molecule, BCAM) were detected exclusively in 3D cultured cells while CD71 (i.e., transferrin receptor protein, TFRC), CD91 (i.e., prolow-density lipoprotein receptor-related protein 1, LRP1) and CD280 (i.e., c-type mannose receptor 2, MRC2) were found to be significantly upregulated in 3D cultured NCI-H23 cells (Table 1). Correspondingly, CD73 (i.e., 5′-nucleotidase, NT5E), CD118 (i.e., leukemia inhibitory factor receptor, LIFR) and CD228 (i.e., melanotransferrin, MELTF) were exclusively identified in 2D cultured cells whereas, CD49b (i.e., integrin alpha-2, ITGA2), CD49f (i.e., integrin alpha-6, ITGA6) and CD54 (i.e., intercellular adhesion molecule 1, ICAM1), CD147 (i.e., basigin, BSG) and CD221 (i.e., insulin-like growth factor 1 receptor, IGF1R), were found upregulated in 2D cultured NCI-H23 cells (Table 2).

**Table 1:** Top 10 most abundant cell-surface proteins found upregulated or solely expressed/detected in 3D-cultured NCI-H23 cells

UniProt Acc #	Gene	Protein description	3D culture regulation	Expression in tumor	Expression reference
P50895	BCAM	Basal cell adhesion molecule (CD239)	Detected in 3D only	Stroma	J Natl Cancer Inst. 2015; 107:djv211.
Q6YHK3	CD109	CD109 antigen	Detected in 3D only	Stroma	J Proteomics. 2012; 77:87–100.
P14209	CD99	CD99 antigen	Detected in 3D only	Stroma	Int J Cancer. 2012; 131:2264–73.
P43121	MCAM	Cell surface glycoprotein MUC18 (CD146)	Detected in 3D only	Stroma	J Hematol Oncol. 2017; 10:76.
P18433	PTPRA	Receptor-type tyrosine-protein phosphatase alpha	Detected in 3D only	Stroma	Histochem Cell Biol. 1999; 111:399–403.
Q9UBG0	MRC2	C-type mannose receptor 2 (CD280)	Upregulated in 3D	Stroma	Clin Exp Metastasis. 2016; 33:151–65.
Q07954	LRP1	Prolow-density lipoprotein receptor-related protein 1 (CD91)	Upregulated in 3D	Stroma	Clin Cancer Res. 2011; 17:2426–33.
P02786	TFRC	Transferrin receptor protein 1 (CD71)	Upregulated in 3D	Parenchyma	Exp Mol Pathol. 2020; 112:104360.
Q03405	PLAUR	Urokinase plasminogen activator surface receptor (CD87)	Upregulated in 3D	Stroma	BMC Cancer. 2014; 14:269.
P10586	PTPRF	Receptor-type tyrosine-protein phosphatase F	Upregulated in 3D	Stroma	Am J Pathol. 2015; 185:1600–9.

**Table 2:** Top 10 most abundant cell-surface proteins found upregulated or solely expressed/detected in 2D-cultured NCI-H23 cells

UniProt Acc #	Gene	Protein description	2D culture regulation	Expression in tumor	Expression reference
P21589	NT5E	5′-nucleotidase (CD73)	Detected in 2D only	Parenchyma	BMC Cancer. 2020; 20:411.
P29323	EPHB2	Ephrin type-B receptor 2	Detected in 2D only	Parenchyma	Tumour Biol. 2017; 39:1010428317691000.
P42702	LIFR	Leukemia inhibitory factor receptor (CD118)	Detected in 2D only	Parenchyma	Cancer Res. 2007; 67:2131–40.
P08582	MELTF	Melanotransferrin (CD228)	Detected in 2D only	Stroma	Exp Cell Res. 2007; 313:2910–19.
P41440	SLC19A1	Folate transporter 1	Detected in 2D only	Parenchyma	Oncotarget. 2018; 9:16807–21.
P35613	BSG	Basigin (CD147)	Upregulated in 2D	Parenchyma	Oncotarget. 2018; 9:26431–52.
P08069	IGF1R	Insulin-like growth factor 1 receptor (CD221)	Upregulated in 2D	Parenchyma	Sci Transl Med. 2019; 11:eaaw7999.
P17301	ITGA2	Integrin alpha-2 (CD49b)	Upregulated in 2D	Parenchyma	Gene. 2018; 643:74–82.
P23229	ITGA6	Integrin alpha-6 (CD49f)	Upregulated in 2D	Parenchyma	Mol Ther Nucleic Acids. 2019; 18:774–86.
P05362	ICAM1	Intercellular adhesion molecule 1 (CD54)	Upregulated in 2D	Parenchyma and Stroma	Virchows Arch. 1996; 428:21–27.

Annotations accessible

### Pathway analysis revealed differential activation/enrichment of metabolic pathways and protein networks in culture dependent manner

**Figure 3:**
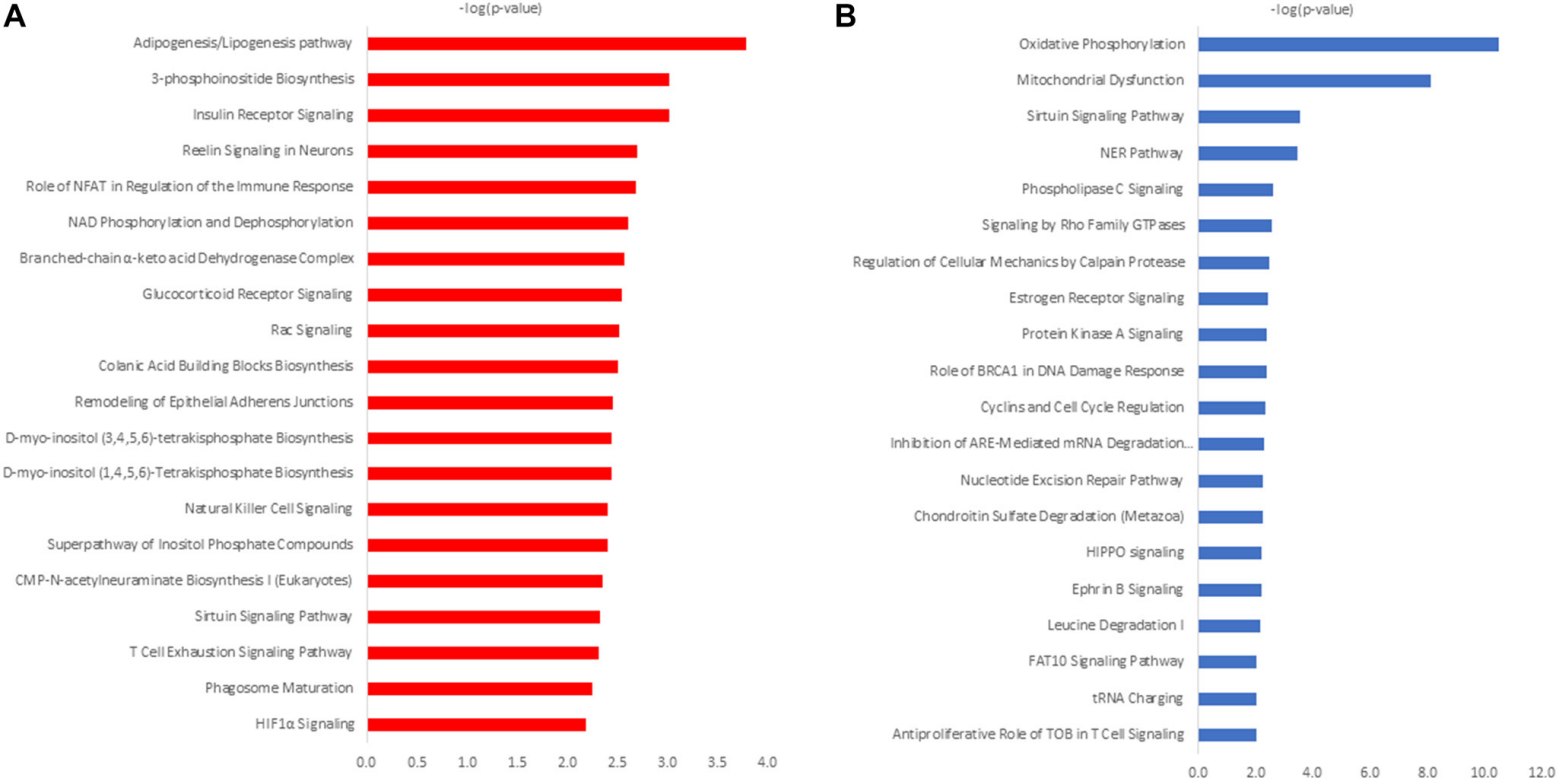
IPA canonical pathway analysis of differentially expressed proteins in 3D cultured NCI-H23 cells (**A**) and 2D-cultured NCI-H23 cells (**B**).

To explore the biological significance of differential proteomic analysis and to prioritize and select cross-validation targets, a subset of membrane proteins detected exclusively and/or found dysregulated in NCI-H23 cells grown in 3D- and 2D-culture were subjected to the Ingenuity^®^ Pathway Analysis (IPA^®^) [QIAGEN Redwood City, https://www.qiagen.com/ingenuity]. Canonical pathway analysis revealed differential activation of metabolic pathways in a culture-dependent manner (Figure 3). While the adipogenesis/lipogenesis pathway (*p*-value 1.63E-04) is the most enriched pathway in 3D cultured cells (Figure 3A), the oxidative phosphorylation (*p*-value 2,93E-11) is the top activated pathway in 2D grown in NCI-H23 cells (Figure 3B). IPA’s biological function analysis revealed the enrichment of top biological functions in a culture dependent manner (Supplementary Table 15) depicting Connective Tissue Development (*p*-value range 2.79E-03 – 1.19E-07) as the most enriched biological function in 3D-culture (Supplementary Table 15A), whereas Organismal Survival (*p*-value range 6.93E-09–3.99E-09) was the most enriched in 2D cultured NCI-H23 cells (Supplementary Table 15B).

Cluster of differentiation (CD) molecules at the cell surface play critical role in cancer immunology (e.g., tumor antigen recognition) [27], tumor biology (e.g., signal transduction and cell migration) [28], tumor microenvironment (e.g., angiogenesis) [29] and cancer immunotherapy therapy (e.g., non-Hodgkin lymphoma) [30]. Therefore, we focused specifically on CD molecules found differentially regulated in a culture-dependent manner as depicted in the IPA’s biological function analysis (Supplementary Table 15). Of these, CD87 (PLAUR), CD91 (LRP1), CD109 antigen, and CD280 (MRC2) were found to be involved in connective tissue growth and fibroblast migration in 3D cell culture (Supplementary Table 15A), whereas CD73 (NT5E), CD118 (LIFR) and CD221 (IGIFR) were found to involved in morbidity and organismal death in NCI-H23 cells grown in 2D culture (Supplementary Table 15B). The enrichment/activation of connective tissue development function and exclusive expression of CD87, CD91, CD109, and CD280 by NCI-H23 cells in 3D culture are in direct agreement with the ability of 3D cultured cells to phenotypically mimic and begin to recapitulate the tumor microenvironment [8].

Next, we explored results of the network analysis by aiming at the networks containing CD molecules. Unlike canonical pathway and/or disease/function analyses that group molecules based on known commonalities or what is accepted in the field, the network analysis groups clusters molecules into networks based on any direct or indirect biological relationship described in the literature [11, 23, 31]. Top identified networks are ranked by statistical significance and number of interacting proteins using Fisher’s exact test [32].

The IPA’s network analysis showed enrichment of 25 functional protein networks in NCI-H23 cells grown in 3D and 2D cell culture (Supplementary Table 16). Based on the involvement of CD molecules in a given network, top disease/functions in 3D cultured cells (Supplementary Table 16A) include: CD146 (MCAM)-cancer and angiogenesis, network #2, CD99-cellular growth and proliferation, network #6, CD280 (MRC2)-organization and proliferation, network #7, CD87 (PLAUR) and CD109-cancer, network #8, CD91 (LRP1)-organization and angiogenesis, network #10 and CD239 (BCAM)-connective tissue disorder and angiogenesis, network #17 (Supplementary Figures 2–7). In 2D-cultured cells (Supplementary Table 16B), networks containing CD molecules include: CD73 (NT5E)-cancer and metastasis, network #4, CD221 (IGF1R)-developmental disorder, network #8, CD118 (LIFR)-metastasis and chemotaxis, network #13 and CD228 (MELTF)-cellular growth, network #14, are depicted in Supplementary Figures 8–11.

**Figure 4:**
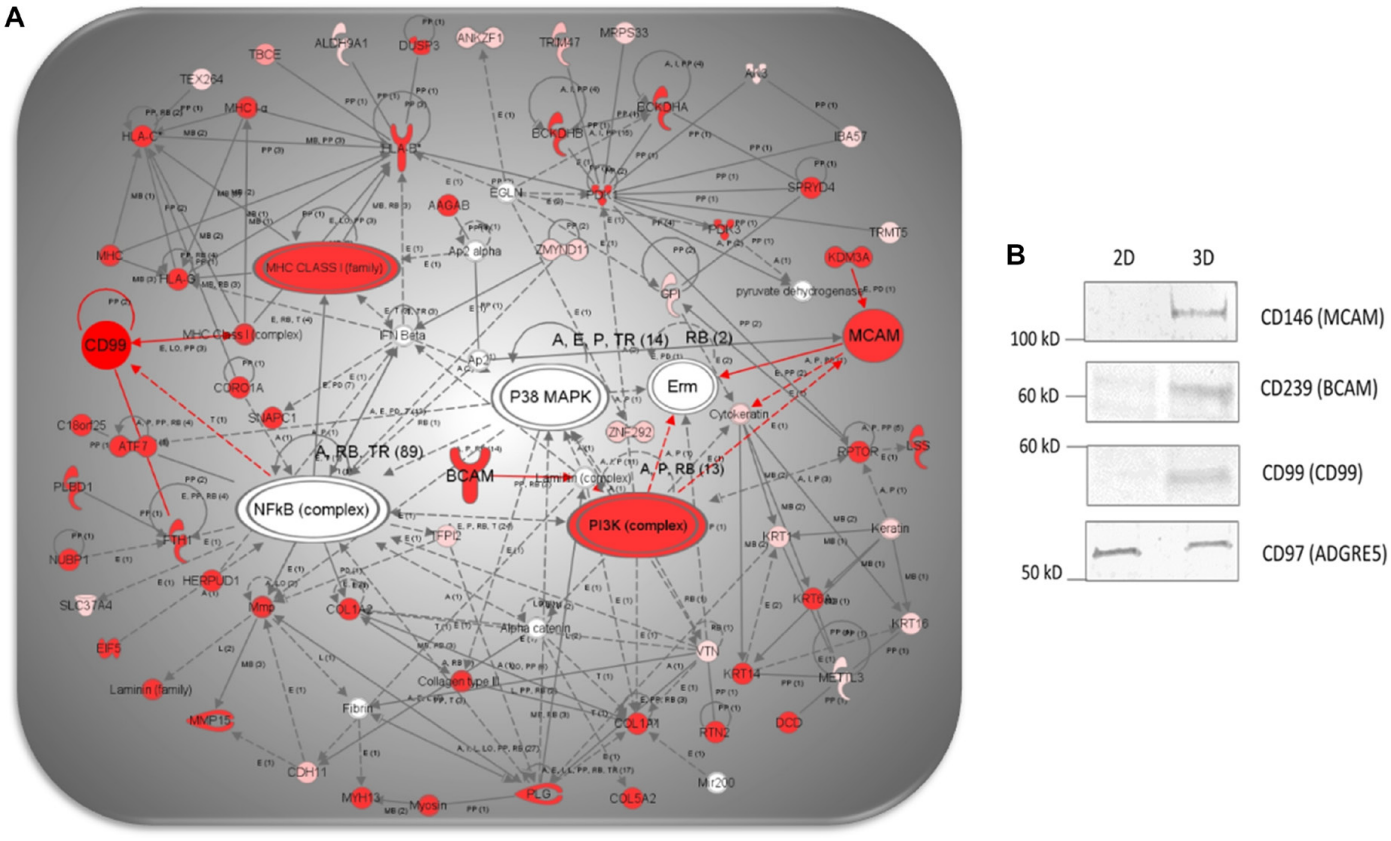
(**A**). IPA network analysis of differentially expressed proteins showing a merged network generated from overlapping networks 2, 6 and 17, featuring CD99, CD239 and CD146, depicting direct (i.e., full red lines) and indirect (i.e., dotted red lines) interactions (i.e., highlighted in red), previously described in the literature. (**B**) Comparative WB analysis showing cropped images of CD99, CD239 and CD146 in 3D- and 2D-cultured NCI-H23 cells.

Moreover, the network analysis also revealed that cancer and angiogenesis, network #2, cellular growth and proliferation, network #6, and connective tissue disorder and angiogenesis, network #17 represent overlapping networks in 3D cultured cells, indicating their interaction at functional, signaling and biological level. The merged network analysis (Figure 4A) depicts PI3K complex, NFkB complex, P38 MAPK and IFN-beta as the most prominent signaling nodes, displaying direct and/or indirect interactions with all of the three CD molecules (i.e., CD99, CD146, CD239) we selected for validation. Taken together, these findings further corroborate the importance and distinctive culture-dependent regulation of CD molecules in 3D-cultured NCI-H23 cancer cells.

### Western blot analysis confirmed exclusive detection of CD146, CD99, and CD239 in 3D cultured NCI-H23 cells

Based on results obtained using statistics, PANTHER bioinformatics, IPA analysis, their role in tumor biology (i.e., angiogenesis, proliferation, metastasis) and the availability of commercial WB antibodies, we selected CD146, CD99, and CD239, identified exclusively in 3D cultured cells, for orthogonal validation using WB.

The human CD146, also known as the melanoma cell adhesion molecule and/or the cell surface glycoprotein MUC18, is encoded by the MCAM gene [33]. CD146 plays a role in cell adhesion, particularly in vascular tissue. The human protein atlas (HPA) [34] showed that at the RNA level CD146 exhibits low specificity for normal/healthy tissue and was detected in many organs including lung. However, at the protein level CD146 is detected highly expressed only in normal cerebral cortex, soft and adipose tissue. Medium expression was observed in normal kidney, adrenal gland placenta breast and tonsils. Interestingly, CD146 was not found expressed in normal lungs at the protein level [34].

In human cancers, CD146’s RNA was detected in many malignancies with the highest levels found in melanoma and renal cancer, and lower level in lung cancer and colorectal cancer, according to the cancer genome atlas (TCGA). At the protein level, immunohistochemistry (IHC) revealed similar pattern depicted by distinct membranous CD146 staining in majority of patients with malignant melanomas and head and neck cancers, whereas lung cancer and endometrial cancer represent a smaller group with lower CD146 expression level [34].

In cell lines, in accordance to the human protein atlas (HPA) accessible consensus normalized expression (“NX”) values, the highest level of CD146’s RNA was observed in endothelial (i.e., HUVEC/TERT2, NX-value: 125.3), skin (i.e., TIME, NX-value:122.3), and melanoma (i.e., WM-115, NX: 97.3) cells. Lower CD146 expression was found in sarcoma (i.e., U-2OS, NX: 17.9), breast cancer (i.e., EFO-21, NX: 10.7) and very low level in colon cancer (i.e., CACO-2, NX: 1.9) and lung cancer (i.e., SCLC-21H, NX 1.4; A569, NX:0.5). Interestingly, CD146 was found to play a critical role in the migration and proliferation in the human pulmonary large cell neuroendocrine carcinoma [35]. However, there is no record in HPA showing the CD146 expression in the NCI-H23 cells at the RNA and/or protein level [34]. The results of comparative WB analysis depicted in Figure 4B is concordant with the results of the LC-MS analysis showing exclusive identification of CD146 at the cell surface of 3D grown NCI-H23 cells. However, CD146 was previously detected by flow cytometry in A549 NSCLC cell line, harboring G12S homozygous KRas mutant, grown in 2D culture under oxidative stress [36]. For that reason, we wanted to ascertain the status of MCAM gene at the transcriptional level in 2D-cultured NCI-H23 cells. We carried out RNA ISH assay (i.e., RNAscope 2.5 HD) [37]. The analysis showed low to moderate level of MCAM expression at the RNA level in most of 2D cultured NCI-H23 cells (Supplementary Figure 13), suggesting that MCAM transcription is present in 2D culture.

The human CD99 antigen, also known as the protein MIC2, is a cell surface glycoprotein encoded by the pseudo-autosomal gene MIC2 [38]. At the RNA level, CD99 is detected in all tissues with highest in level brain, female tissues and muscles. However, at the protein level CD99 was detected highly expressed in normal bone marrow, lymphoid tissues, upper digestive tract, female tissues, and male tissues. Medium expression was observed in normal kidney, adrenal gland and skin, whereas lungs, adipose tissue and colon showed low expression level [34].

CD99’s RNA has been detected in many malignancies in accordance to TCGA. CD99 showed the highest transcription level in malignant glioma and melanoma and lower level in lung cancer, pancreatic cancer, breast cancer and others. At the protein level, high CD99 expression has been observed in Ewing sarcoma, lymphoblastic lymphoma and malignant glioma whereas liver cancer and pancreatic cancer represent a smaller group with low CD99 expression [34].

In cell lines, the high level of CD99 at the transcriptional RNA level was observed in sarcoma (i.e., ASC diff, NX-value: 124.2), foreskin fibroblasts (i.e., BJ hTERT, NX-value:100.3), and malignant glioma (i.e., U138 MG, NX-value: 49.6) cells. Lower CD99 expression was found in lung cancers (i.e., A549, NX-value: 27.4; HBEC3-KT, NX-value: 18.1), among others. Interestingly, there is no record in HPA showing the CD99 expression in the NCI-H23 cells at the RNA level [34]. The results of our WB analysis depicted in Figure 4B. are concordant with the results of the LC-MS analysis depicting exclusive identification of CD99 in 3D grown NCI-H23 cells.

The human CD239, also known as the basal cell adhesion molecule and/or the Lutheran antigen, is a cell surface glycoprotein which is encoded by the BCAM gene [39]. CD239 is a member of the immunoglobulin superfamily. It is a receptor for the extracellular matrix protein laminin, involved in tumor metastasis.

At the RNA level, CD239 has been detected in many tissues with highest expression in kidney, thyroid gland and female tissues. At the protein level CD239 has been found highly expressed in normal kidney, nasopharynx, fallopian tube and placenta. Medium expression was observed in normal lungs, colon, gallbladder, breast and heart muscle, whereas normal cerebellum, thyroid gland, duodenum, liver and pancreas show low CD239 expression levels [34].

CD239’s RNA has been detected in many malignancies in accordance to TCGA. It showed the highest transcriptional level in ovarian cancer, endometrial and prostate cancer and lower level in lung cancer, breast cancer urothelial cancer and others. At the protein level, high CD239 expression has been observed in breast cancer, ovarian cancer prostate cancer whereas colorectal cancer, pancreatic cancer and urothelial cancer showed medium expression level. Lung, stomach and liver cancer represent a group with low CD239 expression [34].

In cell lines, the high RNA level of CD239 expression has been observed in skin (i.e., HaCaT, NX-value: 62.8), breast cancer (i.e., SK-BR-3, NX-value: 58.9). Lower CD239 expression was found in lung cancer (i.e., A549, NX-value: 27.4; HBEC3-KT, NX-value: 18.1), colon cancer (i.e., CACO-2, NX-value: 14.2) among other cell lines. Significantly, there is no record in HPA showing the CD239 expression in the NCI-H23 cells at the transcriptional RNA level [34]. The results of our WB analysis are in agreement with the LC-MS confirming exclusive detection of CD239 in 3D grown NCI-H23 cells (Figure 4B).

### Label-free quantitation confirmed upregulation of total Ras, K-Ras4B^WT^ and K-Ras4B^G12C^ mutant in 3D-cultured NCI-H23 cells

**Figure 5:**
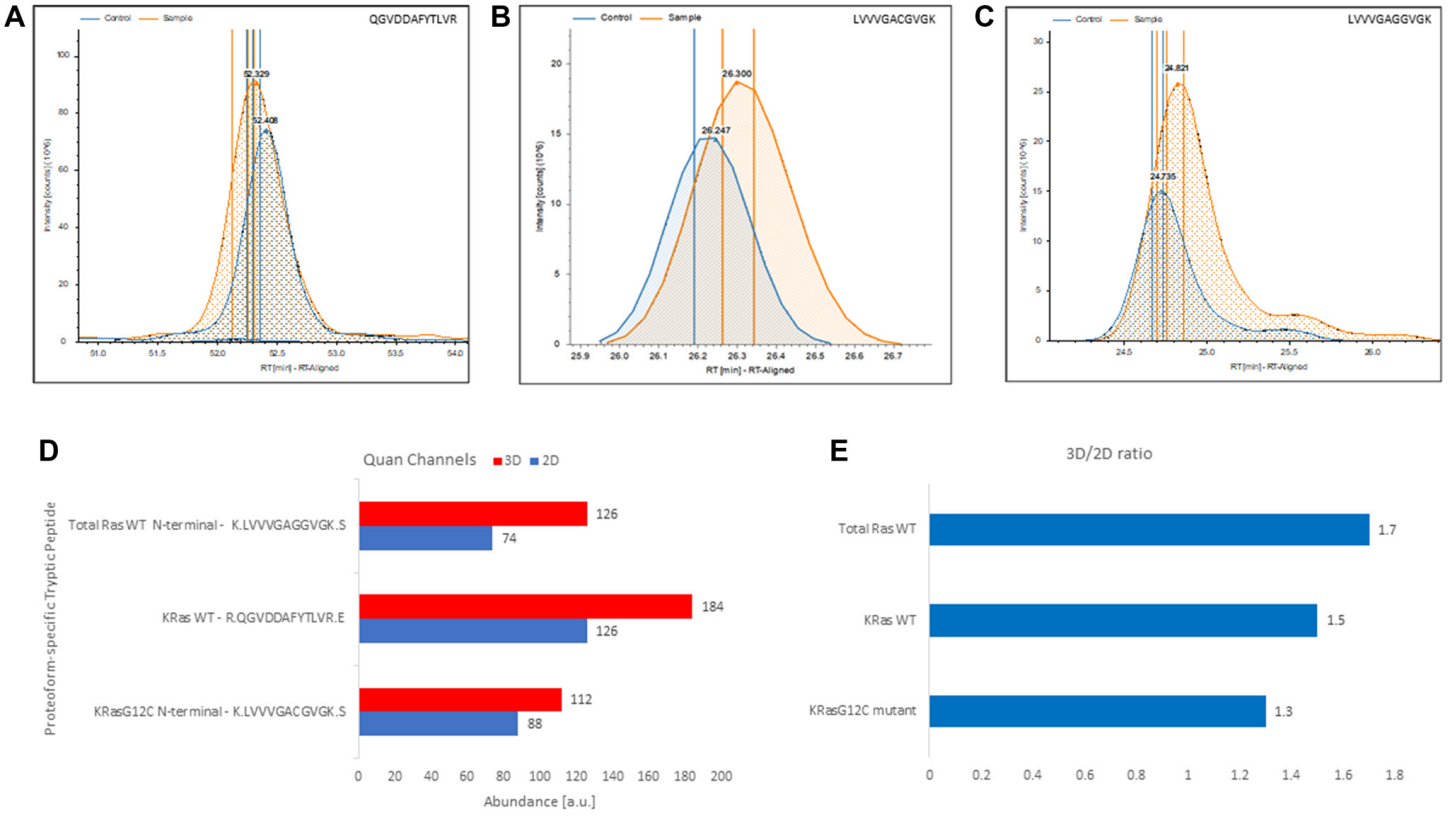
Label-free, gel-free, and antibody-free quantitation of Ras from complex mixture targeting exclusively proteoform-specific tryptic peptides detected in 3D-cultured and 2D-cultured NCI-H23 cells. (**A**) Extracted ion chromatograms of the KRas4B^WT^ proteoform-specific tryptic peptide QGVDDAFYTLVR identified in control (i.e., 2D cultured NCI-H23 cells) and the sample (i.e., 3D-cultured NCI-H23 cells). (**B**) Extracted ion chromatograms of the KRas4B^G12C^ allele-specific N-terminal mutant tryptic peptide LVVVGACGVGK identified in control (i.e., 2D cultured NCI-H23 cells) and the sample (i.e., 3D-cultured NCI-H23 cells). (**C**) Extracted ion chromatogram of total Ras^WT^ specific N-terminal LVVVGAVGVGK peptide identified in control (i.e., 2D cultured NCI-H23 cells) and the sample (i.e., 3D-cultured NCI-H23). (**D**) 3D and 2D quantitative channels readings for the total Ras^WT^ specific N-terminal LVVVGAVGVGK peptide, the KRas4B^WT^ proteoform-specific tryptic peptide QGVDDAFYTLVR and the KRas4B^G12C^ allele-specific N-terminal mutant tryptic peptide LVVVGACGVGK, respectively. (**E**) Calculated 3D/2D ratios for total Ras^WT^, KRas4B^WT^, and KRas4B^G12C^ proteoforms detected in membrane fraction of NCI-H23 cells grown in 3D and 2D culture, respectively.

Recently, CD239 was found to play a functional role in the metastatic spreading of monoallelic KRAS-mutant driven colorectal cancer by mediating interactions between the tumor and its microenvironment [40]. The expression and localization of CD239 was validated by immunohistochemistry in preclinical monoallelic KRAS mutant mouse model of hepatic metastasis [40]. In 3D-cultured NCI-H23 bearing the monoallelic KRAS mutant, we confirmed exclusive CD239 expression using WB. Notably, our spectral counting-based measurements utilizing total tryptic PSM counts showed significant upregulation of Ras proteoforms in a culture dependent manner (Supplementary Table 9). Therefore, we wanted to verify these measurements using label-free quantitation by the exclusive targeting of KRas proteoforms- and allele specific tryptic peptides (i.e., KRas4B^WT^, QGVDDAFYTLVR; KRas4B^G12C^, LVVVGACGVGK), NRas (i.e., NRas^WT^, SFADINLYR) and a total Ras (i.e., Ras^WT^, LVVVGAVGVGK) shown in Supplementary Tables 3–4 [41]. Extracted ion chromatograms shown in Figure 5A–5C depict relative abundance of KRas4B^WT^, KRas4B^G12C^ and total Ras. This analysis, unequivocally confirmed upregulation of KRas4B^WT^, KRas4B^G12C^ and a total Ras^WT^ in 3D-culture (Figure 5D–5E). These results allow us to hypothesize that CD239 may have a role in metastatic spreading of NSCLC similar to the one in the context of colon cancer harboring monoallelic KRas mutants [40].

## DISCUSSION

Lung cancer is the leading cause of cancer-related deaths in the USA and worldwide [1]. Yet, approximately 95% of new drug candidates validated in preclinical phase eventually fail in clinical trials [42]. This high failure rate is generally attributed to the inability of the commonly used two-dimensional (2D) cancer cell culture to recapitulate native three-dimensional (3D) growth of malignant cells in human tumors [5]. In particular, 2D culture does not mimic the natural 3D tumor microenvironment critical for tumor growth and survival [43]. Consequently, false positive compounds regularly qualify for clinical trials, leading to high attrition rates and a waste of time and money. To address these shortcomings, multiple preclinical testing models have been developed to better simulate the natural *in vivo* conditions. One such model is 3D cell culture that has been proposed as a more accurate and relevant preclinical testing model, not only biomechanically but also at the genomic, proteomic, and metabolomic level [25].

MS-based proteomics has been increasingly used in cell-surface profiling due to the limitation of genomic data to provide explicit information about cell surface protein expression level, actual post-translational modification status and/or explicit subcellular location of a given target [44]. Our laboratory has pioneered and applied proteomics for profiling of the cell-surface proteome in cell lines and tissue specimens [11, 19, 21–23, 45, 46]. Despite the importance of preclinical testing models for lung cancer drug target and biomarker discovery, there are no reports to date on in-depth comparative proteomic profiling of any lung cancer cell lines grown in 3D vs. 2D culture. Recent advances in cancer immunotherapy involving the targeting of proteins at the surface of cancerous cells, further emphasize the need for comprehensive mapping of the lung cancer cell surface proteome and development of approaches which are capable of in-depth profiling of the surface of *in vitro* cultured cancer cells [23, 47]. Our investigation begins to address the basic shortage of differential molecular profiles/maps of preclinical lung cancer testing models with the ultimate goal to facilitate evidence-based drug target and biomarker testing and/or discovery.

To obtain a detailed map and differential molecular profile of the crude membrane proteome isolated from NSCLC preclinical model cell line, we applied shotgun proteomics to analyze microsomal fractions from the NCI-H23 cells grown in 3D- and 2D-culture. This analysis uncovered a total 334 protein species unique to 3D-cultured cells, 598 unique to 2D cultured cells and a total 234 common protein species found significantly dysregulated under both culture conditions, revealing a non-redundant map/catalogue containing a total 1,166 proteins regulated in a culture dependent manner (Supplementary Table 12), representing 24% of all non-redundant protein species identified in this study. This may explain why false positive compounds often enter clinical trials. Importantly, this molecular map provides detailed qualitative and distinct quantitative information on proteins differentially regulated in a culture-dependent manner. This catalogue enables reasonable selection and ranking of putative targets based on their detectability and relative abundance estimated by LC-MS.

Regardless of a cell culture type, cancer cells require a continuous supply of energy and structural components to support their growth and proliferation [48]. Due to persistent tumor hypoxia, cancer cells are forced to adapt their bioenergetic pathways to survive and proliferate in harsh hypoxic tumor microenvironment [49]. In comparisons to non-transformed healthy cells, transformed malignant cells possess remarkable metabolic flexibility characterized primarily by the ability upsurge glycolysis and repress oxidative phosphorylation (i.e., Warburg effect) [50], in order to secure cell survival and growth in hypoxia [49]. The principal regulator of intensified glycolysis is the hypoxia inducible factor 1 (HIF-1), a transcription factor responsible for increased transcription/translation of glycolytic enzymes induced by hypoxia [51]. In comparison with 2D cultured cells, 3D-cultured cells are exposed to a much higher level of hypoxia and/or anoxia, featuring oxygen gradients which exist in the tumor microenvironment [52, 53]. Accordingly, the PANTHER pathway analysis revealed activation of hypoxia response via HIF and glycolysis pathway in 3D cultured NCI-H23 cells (Figure 2B). Correspondingly, the TGF-beta signaling was found highly activated in 2D culture, which is consistent with the role of this pathway in stimulating mitochondrial oxidative phosphorylation in the absence and/or low level of hypoxia present in 2D culture [52].

Also, it is widely recognized that tumors can switch to lipid-dependent metabolism for energy supply, employing adipogenesis/lipogenesis pathway for de novo synthesis of endogenous lipids and/or utilize the exogenous lipids from tumor microenvironment [54]. Significantly, lipogenesis-driven *de novo* synthesis of fatty acids (FA) is linked to oncogene signaling, responsible for endothelial cell recruitment and induction of tumor angiogenesis in response to hypoxia [55]. The IPA canonical pathway analysis, revealed the adipogenesis/lipogenesis pathway as the most enriched pathway in 3D cultured cells (Figure 3A), suggesting that hypoxia-driven activation of *de novo* FA synthesis in 3D cultured NCI-H23 cells is taking place in order to initiate endothelial cell recruitment and induction of neo-angiogenesis [56]. In addition, canonical pathway analysis exposed the activation of HIF1-alpha signaling in 3D culture (Figure 3A), indicative of hypoxia-driven glycolysis [51]. Significantly, it is now widely recognized that adipogenesis/lipogenesis pathway is closely linked to glycolytic pathway [57]. In 2D-cultured cells, the IPA canonical pathway analysis exposed the oxidative phosphorylation pathway as a top significantly enriched pathway (Figure 3B). This is in agreement with findings showing that oxidative phosphorylation is predominant type of energy metabolism in lung cancer cell lines grown in 2D-culture [49]. Taken together, the canonical pathway analysis is indicative of the ability of shotgun proteomics to capture broad phenotypical changes, depicting differential activation of principal metabolic pathways, in a culture-dependent manner, indicating that 3D-cultured cells may be better than 2D-cultured cells in mirroring hypoxia, an important tumor feature that drives drug resistance *in vivo* [52].

The IPA disease and biological function analysis revealed connective tissue development as the top biological function (Supplementary Table 15), exemplified by the enrichment of multiple fibroblast functions and connective tissue growth, indicative of 3D cultured cells ability to sense 3D milieu and initiate phenotypical changes necessary for the creation of a tumor microenvironment that is critical for tumor survival [43]. Importantly, this analysis revealed a subset of CD molecules involved in this phenotypical transformation, showing exclusive expression of CD109 and CD146 in 3D culture, and upregulation of CD87, CD91 and CD280 in a culture dependent-manner (Table 1). This remarkable phenotypical transformation captured by shotgun membrane proteomics is equivalent to hypoxia-induced vascular mimicry in melanoma [58, 59] that we previously investigated using similar approach [45].

Next, we focused specifically on differentially regulated CD molecules/antigens, primarily due to their importance in cancer diagnostics (i.e., cancer biomarkers)[60, 61], cancer treatment (i.e., cancer immunotherapy)[62, 63] and our interest in the cell surface proteomics of cancer cell lines [23] and tumor xenografts [11] harboring KRas mutants in the context of biomarker and drug target discovery [47]. We were the first show colocalization of CD147 (BSG) and CD318 (CDCP1) at the surface of cancer cell line expressing KRas4B mutant [23]. Importantly, subsequent investigations established CD318 as a viable target for radiological staging and treatment of pancreatic cancer [47, 64].

In this study, we confirmed exclusive expression of CD146, CD99 and CD239 on the surface of 3D-grown NCI-H23 cells (Figure 4B) and showed their potential interaction through the activation of protein networks facilitated by PI3K, NFkB, and IFN-beta signaling (Figure 4A). A recent study focused on the evaluation of the KRas^G12C^ direct covalent inhibitor ARS1620 using patient-derived xenograft (PDX) models and found that intrinsic resistance of NSCLC to ARS1620 monotherapy, driven by nongenetic adaptive mechanisms, can be alleviated by concomitant application of PI3K inhibitors [65]. This study also showed that the PI3K pathway is not under the exclusive control of the KRas^G12C^ mutant in NSCLC PDX models. Based on the IPA network analysis (Figure 4A), and WB validation, it is tempting to speculate that CD146, CD99 and CD239 are downstream PI3K effectors involved in pro-angiogenic [66] malignant stroma [67] regulated metastasis [40] and may be explored as potential immunotherapeutic cell surface targets in the context of KRas^G12C^ driven NSCLC.

In this investigation, both the LC-MS and the WB analysis unambiguously established the presence/expression of CD146 in 3D-culture, whereas both LC-MS and WB were negative for the CD146 expression in 2D-cultured NCI-H23 cells (Figure 4B). However, the RNAscope ISH assay was clearly positive for the presence of MCAM transcripts in 2D-cultured NCI-H23 cells (Supplementary Figure 12). It is well accepted that, with the exception for highly abundant housekeeping proteins, mRNA levels correlate poorly with protein level in cultured cell lines and tissues [68]. Furthermore, cell surface proteins show the worse correlation between protein and RNA abundance primarily because of complex posttranscriptional mechanisms controlling their expression (e.g., translation rate, degradation rate, transport) [69]. Importantly, our LC-MS and WB analyses were carried out using the membrane fraction, and it is possible that a very low level of ubiquitinated CD146 might exist in the cytosol, undergoing rapid degradation [68]. These results underscore the importance of direct proteomic analyses to identify/quantify deferentially expressed cell surface proteins in cancer cell lines or tumor tissue [44]. Significantly, a tissue based IHC study targeting CD146 showed that CD146 expression was clearly predictive of lymph node metastasis in patients with NSCLC (*n* = 118) [70]. Another tumor tissue based study, relying on well-characterized clinical cohorts, reveled CD99 as a novel clinically applicable NSCLC prognostic stromal marker [67]. Similarly, gene expression profiles obtained from 1106 NSCLC tissue specimens, revealed BCAM (CD239) as a part of stromal biomarker panel for early-stage NSCLC survival stratification [71].

Taken together, the results of these investigations are indicative of the ability of 3D-cultured cells to faithfully recapitulate tumor-like phenotype, and the efficacy of comparative shotgun proteomics in capturing these changes. Phenotypically these changes at the proteome level are consistent with stroma-like transformation of epithelial NCI-H23 cells in 3D culture, where uniquely expressed CD molecules play important role. Specifically, CD146, CD99 and CD239 which may be considered as therapeutic targets and would not have been discovered employing conventional 2D-culture.

The NCI-H23 cells harbors KRas4B^G12C^ homozygous mutant and could be used as a preclinical NSCLC model for testing allele-specific covalent inhibitors that bind to the cysteine at position 12 of the G12C KRas4B mutant [72]. In this study, we unambiguously showed that 3D culture induces upregulation of both wild type and mutant KRas4B allele in NCI-H23 cells (Figure 5). Importantly, the present approach allows for direct proteoform- and allele-specific quantitation of changes in KRas^WT^ and KRas^G12C^ mutant regulation, and can be easily employed in quantifying responses to allele-specific covalent inhibitors or any other therapeutic compounds affecting Ras expression [73]. To our knowledge, this is the first report of gel-free and antibody-free bottom-up quantitative LC-MS analysis of KRas targeting exclusively proteoform-specific tryptic peptides. The shotgun proteomics is also amenable to absolute antibody-free quantitation of Ras isoforms using synthetic heavy isoform-specific tryptic peptide standards for LC-MS-based parallel reaction monitoring (PRM), we previously described targeting xenotropic and polytropic retrovirus receptor 1 (XPR1) in a complex membrane protein mixture [23]. Importantly, this approach allows for concomitant hypothesis-free proteomic profiling and the PRM-based quantitation applied on the same sample, as previously described [23]. This would greatly facilitates reduction in the variability associated with multiple sample preparations.

## MATERIALS AND METHODS

### Materials

Ammonium bicarbonate (NH_4_HCO_3_), phenylmethylsulfonyl fluoride (PMSF), formic acid (HCOOH), and iodoacetamide (IAA) were obtained from Sigma (St. Louis, MO). Tris[2-carboxyethyl] phosphine (TCEP) Bond-Breaker^™^ was from Pierce (Rockford, IL). Acid-cleavable surfactant: 3-[3-(1,1-bisalkyloxyethyl)pyridin-1-yl]propane-1-sulfonate (PPS) was from Protein Discovery Inc. (Knoxville, TN). All chemicals used were A.C.S. grade or higher, and all solvents used were HPLC-grade or higher. Sequence-grade modified trypsin was obtained from Promega (Madison, WI). The anti-MCAM (CD146), anti-BCAM (CD239), anti-CD99, and anti-CD97 antibodies used for western blot (WB) were from Abcam (Cambridge, United Kingdom).

### Cell culture

NCI-H23 cells were obtained from the American Type Culture Collection (ATCC, Rockville, MD). 2D culture was carried out in RPMI 1640 medium supplemented with 8 mM l-glutamine (GIBCO–BRL, Basel, Switzerland) and 5% fetal calf serum (FCS) (Fakola, Basel, Switzerland). 3D culture was carried out using global eukaryotic microcarriers platform from Global Cell Solutions (GCS, Charlottesville, Virginia) as previously described [74]. At the end of the culture process, both 2D and 3D grown cells were detached from the surface using trypsin. About 10^7^ cells were collected and washed with PBS and stored at –80°C.

### Microsomal fraction preparation, digestion, and fractionation

Microsomal fraction was isolated as previously described [23]. Briefly, each cell pellet was thawed in 50 mM NH_4_HCO_3_ containing 1 mM PMSF. Cells were homogenized using tip sonication. After debris removal, the lysate was reduced using 3 mM TCEP and alkylated using 5 mM IAA. Following the ultracentrifugation at 100,000 × g for 1.5 h, pellets containing enriched microsomal fraction were resuspended using tip sonication in 25 mM NH_4_HCO_3_. Next, protein concentration was determined using the BCA Protein Assay Kit (Pierce). Microsomal fraction aliquots, 500 μg each, were lyophilized and then solubilized in 500 μL of 60% CH_3_OH/40% 25 mM NH_4_HCO_3_ (v/v), containing 0.1% PPS, as previously described [21]. After tryptic digestion, samples were desalted, lyophilized and reconstituted in 200 μL of 45% acetonitrile containing 0.1% formic acid. Each digest was then fractionated using SCX chromatography (Supplementary Figure 13) and a total of 12 peptide fractions were collated, as previously described [19].

### LC-MS and raw data analysis

SCX fractions were analyzed in duplicates using an EASY-nLC 1200 System (ThermoFisher Scientific) coupled on-line to an Orbitrap Elite mass spectrometer (ThermoFisher Scientific). Each SCX fraction was reconstituted in 0.1% TFA and 1 μg of peptides loaded onto an EASY-Spray, 25 cm long reversed-phase C18 column (ThermoFisher Scientific). After injection, peptides were eluted using a linear gradient starting with 2% mobile phase B (0.1% formic acid in ACN) to 40% solvent B (0.1% formic acid in 80% ACN). The mass spectrometer was operated in a data-dependent mode, using the peptide *m/z* range of 400−1800, monitored at the resolution of 60,000. Each MS scan was followed by 15 MS/MS scans, wherein the 15 most abundant precursor ions were dynamically selected for collision-induced dissociation using normalized collision energy of 35%. Protein identification was carried out using the SEQUEST-based search against the non-redundant human proteome database (SwissProt release v57.15) utilizing the Proteome Discoverer 2.2 (ThermoFisher Scientific). For the monoisotopic peptide precursor ions (i.e., MS spectra), mass tolerance was set at 5 ppm; and for the fragment ions (i.e., MS/MS spectra), mass tolerance was set at 0.6 Da for fully tryptic peptides, allowing for up to two missed cleavages. Dynamic amino acid modifications were added for the detection of the following: +57.021 Da for carboxyamidomethylated cysteines, +15.994 Da for oxidized methionines and +0.984 deamidated asparagine/glutamine. A strict peptide false discovery rate (FDR) of ≤ 0.01 was set using Percolator-based statistical evaluation [75]. To increase the quality and reliability of protein identifications and enforce economy in the number of identified proteins, protein grouping was employed. Proteome Discoverer 2.2 was used for spectral counting based relative quantitation of changes in protein regulation between 2D and 3D cultured cells utilizing peptide-spectral matching (PSM) readouts computed by the Percolator algorithm [76]. The same software was used for the label-free quantitation of Ras targeting exclusively proteoform-specific peptides.

### Statistics and bioinformatics

Significantly dysregulated proteins were revealed using binomial probability and FDR calculation [77]. PSORT (Horton P) and TMHMM (Krogh, A) algorithms were used to classify and characterize membrane proteins. To map cell surface proteins, we used the mass spectrometric-derived Cell Surface Protein Atlas (CSPA) [24] accessible at https://wlab.ethz.ch/cspa/. The PANTHER (protein annotation through evolutionary relationship) classification system (http://www.pantherdb.org/) was employed to classify significantly enriched protein species and corresponding pathways [78]. Finally, data were analyzed using literature-based Ingenuity Pathway Analysis IPA^®^ (QIAGEN Inc., https://www.qiagenbioinformatics.com/products/ingenuity-pathway-analysis) to generate significantly enriched protein networks and select/prioritize cross-validation targets.

### Western blot analysis

Cells were lysed in 25 mM of NH_4_HCO_3_ buffer supplemented with 1 mM PMSF and homogenized by five cycles of 10-second sonication (20% intensity) using a Bronson microprobe tip-sonicator. The homogenate was centrifuged at 1000 × g for 10 min to remove unbroken cells and cellular debris. The supernatant was ultracentrifuged at (100,000 × g) for 1.5 hrs. using a Beckman MLS50 rotor (Brea, CA, USA). The membrane pellet was resolubilized in 25 mM of NH4HCO3 buffer, and the protein concentration of the solution was determined with the BCA Protein Assay Kit (Pierce). An equal amount of protein was run on SDS-PAGE (Life Technologies). Resolved proteins were transferred onto a 0.2 μm nitrocellulose membrane (Bio-Rad, Hercules, CA, USA), blocked with 5% non-fat dried milk in PBST (PBS with 0.05% Tween), incubated with primary Ab at 4 °C overnight, washed with PBST, and probed with HRP-conjugated secondary Ab (Jackson ImmunoResearch, West Grove, PA). Immunoreactive bands were visualized by colorimetric detection using the Opti-4CN Substrate Kit (Bio-Rad). We used CD97 as WB control as well as membrane protein isolation reproducibility control since the LC-MS analysis showed no changes in CD97 expression between compared cell culture conditions.

### RNAscope

To evaluate mRNA transcription status of MCAM in 2D grown cells, an *in situ* hybridization (ISH) RNA assay, (i.e., RNAscope 2.5 HD –RED), from Advanced Cell Diagnostics Inc., was applied on fixed 2D-cultured NCI-H23 in accordance with manufacturer’s protocol [79].

### Availability of data and materials

The datasets analyzed in the current study are accessible at the https://vmsshare.nist.gov.

## CONCLUSIONS

In this study we generated a cell surface resource/atlas of a preclinical testing model NCI-H23 cell line, providing insight into phenotypical changes at the proteome level unique to each culture type. This study provides for the first time an antibody-free and gel-free, proteomic method to directly quantify proteoform- and allele-specific changes in KRas^WT^ and KRas^G12C^ mutant expression in complex protein mixture using comparative bottom-up proteomics. This resource describes protein species that are found significantly dysregulated in culture-dependent manner as well as proteins unique to 3D- and 2D-culture, which would not have been observed utilizing solely conventional 2D-culture. Capturing culture-dependent changes in protein expression is essential for providing a baseline to which cells can be accurately compared, followed by the application/testing a given drug candidate. In keeping with these concepts, we captured remarkable metabolic changes taking place in culture-dependent manner. Our proteomic resource also contains information on the difference in enrichment of biological functions and protein networks that can be mined to understand the transformation of NCI-H23 cells in 3D-culture consistent with activation of malignant tumor stroma. In particular, interactions between CD molecules mediating networks are key to regulating phenotypical changes under 3D culture. Among the candidate proteins selected for cross validation, unique expression of CD99, CD146 and CD239 in 3D-cultured cells are indicative of the development of malignant stroma (i.e., tumor microenvironment) induced angiogenesis and metastasis, likely triggered by increased hypoxia present in 3D-culture. Hence, our proteomic resource will provide a valuable set of protein targets for future studies and substantiate the advantage of using 3D-culture to bridge the gap between conventional 2D *in vitro* cultures and *in vivo* animal testing models.

The present proof of the principle proteomic strategy should be widely applicable to other patient-derived cancer cell lines. Evidently, each patient-derived cancer cell line represents a phenotype of a single individual tumor/cancer. Therefore, it is critical to carry out comparative proteomic profiling of a large number of patient-derived cancer cell lines (e.g., NCI-60) grown in 2D- and 3D-culture in order to alleviate the heterogeneity issue and obtain comprehensive molecular profile/atlas of preclinical testing models, followed up by investigations focused on the differences ate the proteome level observed after application of selected drugs. Finally, phenotypical differences at the molecular level between 3D- and 2D-cultured NCI-H23 cells described in this investigation, underscores the importance of characterizing the properties of 3D cell models utilized in preclinical testing, in order to reduce high drug attrition rates. In fact, the use of 3D-cultured cells might be a necessary precursor, and perhaps an alternative, to animal models.

## SUPPLEMENTARY MATERIALS





## Abbreviations

2D: Two-dimensional
3D: Three-dimensional
ATCC: American type culture collection
CSPA: Cell surface protein atlas
FCS: Fetal calf serum
FDR: False discovery rate
HPA: Human protein atlas
HR/AM: High resolution/accurate mass
IAA: Iodoacetamide
IPA: Ingenuity pathway analysis
ISH: In situ hybridization
LC-MS: Liquid chromatography-mass spectrometry
NSCLC: Non-small cell lung cancer
PMSF: Phenylmethylsulfonyl fluoride
SCX: Strong cation exchange
PPS: 3-[3-(1,1-bisalkyloxyethyl)pyridin-1-yl]propane-1-sulfonate
TCEP: Tris[2-carboxyethyl] phosphine
WB: Western blot

## References

[1] Siegel R, Naishadham D, Jemal A. Cancer statistics, 2013. CA Cancer J Clin. 2013; 63:11–30. 10.3322/caac.21166. 23335087

[2] Rotow J, Bivona TG. Understanding and targeting resistance mechanisms in NSCLC. Nat Rev Cancer. 2017; 17:637–58. 10.1038/nrc.2017.84. 29068003

[3] Arrowsmith J, Miller P. Trial watch: phase II and phase III attrition rates 2011-2012. Nat Rev Drug Discov. 2013; 12:569. 10.1038/nrd4090. 23903212

[4] Cook D, Brown D, Alexander R, March R, Morgan P, Satterthwaite G, Pangalos MN. Lessons learned from the fate of AstraZeneca’s drug pipeline: a five-dimensional framework. Nat Rev Drug Discov. 2014; 13:419–31. 10.1038/nrd4309. 24833294

[5] Gillet JP, Varma S, Gottesman MM. The clinical relevance of cancer cell lines. J Natl Cancer Inst. 2013; 105:452–58. 10.1093/jnci/djt007. 23434901 PMC3691946

[6] Gazdar AF, Hirsch FR, Minna JD. From Mice to Men and Back: An Assessment of Preclinical Model Systems for the Study of Lung Cancers. J Thorac Oncol. 2016; 11:287–99. 10.1016/j.jtho.2015.10.009. 26723239 PMC4809191

[7] Langhans SA. Three-Dimensional *in Vitro* Cell Culture Models in Drug Discovery and Drug Repositioning. Front Pharmacol. 2018; 9:6. 10.3389/fphar.2018.00006. 29410625 PMC5787088

[8] Nyga A, Cheema U, Loizidou M. 3D tumour models: novel *in vitro* approaches to cancer studies. J Cell Commun Signal. 2011; 5:239–48. 10.1007/s12079-011-0132-4. 21499821 PMC3145874

[9] Justice BA, Badr NA, Felder RA. 3D cell culture opens new dimensions in cell-based assays. Drug Discov Today. 2009; 14:102–07. 10.1016/j.drudis.2008.11.006. 19049902

[10] Moffat JG, Rudolph J, Bailey D. Phenotypic screening in cancer drug discovery - past, present and future. Nat Rev Drug Discov. 2014; 13:588–602. 10.1038/nrd4366. 25033736

[11] Ye X, Luke BT, Wei BR, Kaczmarczyk JA, Loncarek J, Dwyer JE, Johann DJ, Saul RG, Nissley DV, McCormick F, Whiteley GR, Blonder J. Direct molecular dissection of tumor parenchyma from tumor stroma in tumor xenograft using mass spectrometry-based glycoproteomics. Oncotarget. 2018; 9:26431–52. 10.18632/oncotarget.25449. 29899869 PMC5995176

[12] Yue X, Lukowski JK, Weaver EM, Skube SB, Hummon AB. Quantitative Proteomic and Phosphoproteomic Comparison of 2D and 3D Colon Cancer Cell Culture Models. J Proteome Res. 2016; 15:4265–76. 10.1021/acs.jproteome.6b00342. 27696853 PMC5334570

[13] Gęgotek A, Atalay S, Domingues P, Skrzydlewska E. The Differences in the Proteome Profile of Cannabidiol-Treated Skin Fibroblasts following UVA or UVB Irradiation in 2D and 3D Cell Cultures. Cells. 2019; 8:995. 10.3390/cells8090995. 31466340 PMC6770406

[14] Tölle RC, Gaggioli C, Dengjel J. Three-Dimensional Cell Culture Conditions Affect the Proteome of Cancer-Associated Fibroblasts. J Proteome Res. 2018; 17:2780–89. 10.1021/acs.jproteome.8b00237. 29989826

[15] Kim YE, Jeon HJ, Kim D, Lee SY, Kim KY, Hong J, Maeng PJ, Kim KR, Kang D. Quantitative Proteomic Analysis of 2D and 3D Cultured Colorectal Cancer Cells: Profiling of Tankyrase Inhibitor XAV939-Induced Proteome. Sci Rep. 2018; 8:13255. 10.1038/s41598-018-31564-6. 30185973 PMC6125324

[16] Lee SY, Park SB, Kim YE, Yoo HM, Hong J, Choi KJ, Kim KY, Kang D. iTRAQ-Based Quantitative Proteomic Comparison of 2D and 3D Adipocyte Cell Models Co-cultured with Macrophages Using Online 2D-nanoLC-ESI-MS/MS. Sci Rep. 2019; 9:16746. 10.1038/s41598-019-53196-0. 31727937 PMC6856061

[17] Pavelić SK, Markova-Car E, Klobučar M, Sappe L, Spaventi R. Technological Advances in Preclinical Drug Evaluation: The Role of -Omics Methods. Curr Med Chem. 2020; 27:1337–49. 10.2174/0929867326666190711122819. 31296156

[18] Klaeger S, Heinzlmeir S, Wilhelm M, Polzer H, Vick B, Koenig PA, Reinecke M, Ruprecht B, Petzoldt S, Meng C, Zecha J, Reiter K, Qiao H, et al. The target landscape of clinical kinase drugs. Science. 2017; 358:eaan4368. 10.1126/science.aan4368. 29191878 PMC6542668

[19] Blonder J, Chan KC, Issaq HJ, Veenstra TD. Identification of membrane proteins from mammalian cell/tissue using methanol-facilitated solubilization and tryptic digestion coupled with 2D-LC-MS/MS. Nat Protoc. 2006; 1:2784–90. 10.1038/nprot.2006.359. 17406535

[20] Mann M, Kelleher NL. Precision proteomics: the case for high resolution and high mass accuracy. Proc Natl Acad Sci U S A. 2008; 105:18132–38. 10.1073/pnas.0800788105. 18818311 PMC2587563

[21] Ye X, Johann DJ Jr, Hakami RM, Xiao Z, Meng Z, Ulrich RG, Issaq HJ, Veenstra TD, Blonder J. Optimization of protein solubilization for the analysis of the CD14 human monocyte membrane proteome using LC-MS/MS. J Proteomics. 2009; 73:112–22. 10.1016/j.jprot.2009.08.008. 19709643 PMC3159575

[22] Wigelsworth DJ, Ruthel G, Schnell L, Herrlich P, Blonder J, Veenstra TD, Carman RJ, Wilkins TD, Van Nhieu GT, Pauillac S, Gibert M, Sauvonnet N, Stiles BG, et al. CD44 Promotes intoxication by the clostridial iota-family toxins. PLoS One. 2012; 7:e51356. 10.1371/journal.pone.0051356. 23236484 PMC3517468

[23] Ye X, Chan KC, Waters AM, Bess M, Harned A, Wei BR, Loncarek J, Luke BT, Orsburn BC, Hollinger BD, Stephens RM, Bagni R, Martinko A, et al. Comparative proteomics of a model MCF10A-KRasG12V cell line reveals a distinct molecular signature of the KRasG12V cell surface. Oncotarget. 2016; 7:86948–71. 10.18632/oncotarget.13566. 27894102 PMC5341332

[24] Wollscheid B, Bausch-Fluck D, Henderson C, O’Brien R, Bibel M, Schiess R, Aebersold R, Watts JD. Mass-spectrometric identification and relative quantification of N-linked cell surface glycoproteins. Nat Biotechnol. 2009; 27:378–86. 10.1038/nbt.1532. 19349973 PMC2829300

[25] Antoni D, Burckel H, Josset E, Noel G. Three-dimensional cell culture: a breakthrough *in vivo* . Int J Mol Sci. 2015; 16:5517–27. 10.3390/ijms16035517. 25768338 PMC4394490

[26] Musah-Eroje A, Watson S. A novel 3D *in vitro* model of glioblastoma reveals resistance to temozolomide which was potentiated by hypoxia. J Neurooncol. 2019; 142:231–40. 10.1007/s11060-019-03107-0. 30694423 PMC6449313

[27] Selleri L, Smith MW, Holmsen AL, Romo AJ, Thomas SD, Paternotte C, Romberg LC, Wei YH, Evans GA. High-resolution physical mapping of a 250-kb region of human chromosome 11q24 by genomic sequence sampling (GSS). Genomics. 1995; 26:489–501. 10.1016/0888-7543(95)80167-k. 7607672

[28] Ward Y, Lake R, Faraji F, Sperger J, Martin P, Gilliard C, Ku KP, Rodems T, Niles D, Tillman H, Yin J, Hunter K, Sowalsky AG, et al. Platelets Promote Metastasis via Binding Tumor CD97 Leading to Bidirectional Signaling that Coordinates Transendothelial Migration. Cell Rep. 2018; 23:808–22. 10.1016/j.celrep.2018.03.092. 29669286 PMC6574118

[29] Lawler PR, Lawler J. Molecular basis for the regulation of angiogenesis by thrombospondin-1 and -2. Cold Spring Harb Perspect Med. 2012; 2:a006627. 10.1101/cshperspect.a006627. 22553494 PMC3331684

[30] Turtle CJ, Hanafi LA, Berger C, Hudecek M, Pender B, Robinson E, Hawkins R, Chaney C, Cherian S, Chen X, Soma L, Wood B, Li D, et al. Immunotherapy of non-Hodgkin’s lymphoma with a defined ratio of CD8+ and CD4+ CD19-specific chimeric antigen receptor-modified T cells. Sci Transl Med. 2016; 8:355ra116. 10.1126/scitranslmed.aaf8621. 27605551 PMC5045301

[31] Li N, Zhan X. Signaling pathway network alterations in human ovarian cancers identified with quantitative mitochondrial proteomics. EPMA J. 2019; 10:153–72. 10.1007/s13167-019-00170-5. 31258820 PMC6562010

[32] Zhan X, Desiderio DM. Signaling pathway networks mined from human pituitary adenoma proteomics data. BMC Med Genomics. 2010; 3:13. 10.1186/1755-8794-3-13. 20426862 PMC2884164

[33] Lehmann JM, Riethmüller G, Johnson JP. MUC18, a marker of tumor progression in human melanoma, shows sequence similarity to the neural cell adhesion molecules of the immunoglobulin superfamily. Proc Natl Acad Sci U S A. 1989; 86:9891–95. 10.1073/pnas.86.24.9891. 2602381 PMC298608

[34] Uhlén M, Fagerberg L, Hallström BM, Lindskog C, Oksvold P, Mardinoglu A, Sivertsson Å, Kampf C, Sjöstedt E, Asplund A, Olsson I, Edlund K, Lundberg E, et al. Proteomics. Tissue-based map of the human proteome. Science. 2015; 347:1260419. 10.1126/science.1260419. 25613900

[35] Piao Y, Guo H, Qu Z, Zheng B, Gao Y. CD146 promotes migration and proliferation in pulmonary large cell neuroendocrine carcinoma cell lines. Oncol Lett. 2019; 17:2075–80. 10.3892/ol.2018.9830. 30675274 PMC6341587

[36] Yilmazer A. Cancer cell lines involving cancer stem cell populations respond to oxidative stress. Biotechnol Rep (Amst). 2017; 17:24–30. 10.1016/j.btre.2017.11.004. 29276697 PMC5730381

[37] Wang F, Flanagan J, Su N, Wang LC, Bui S, Nielson A, Wu X, Vo HT, Ma XJ, Luo Y. RNAscope: a novel *in situ* RNA analysis platform for formalin-fixed, paraffin-embedded tissues. J Mol Diagn. 2012; 14:22–29. 10.1016/j.jmoldx.2011.08.002. 22166544 PMC3338343

[38] Goodfellow PN, Pym B, Pritchard C, Ellis N, Palmer M, Smith M, Goodfellow PJ. MIC2: a human pseudoautosomal gene. Philos Trans R Soc Lond B Biol Sci. 1988; 322:145–54. 10.1098/rstb.1988.0122. 2907798

[39] Parsons SF, Mallinson G, Holmes CH, Houlihan JM, Simpson KL, Mawby WJ, Spurr NK, Warne D, Barclay AN, Anstee DJ. The Lutheran blood group glycoprotein, another member of the immunoglobulin superfamily, is widely expressed in human tissues and is developmentally regulated in human liver. Proc Natl Acad Sci U S A. 1995; 92:5496–500. 10.1073/pnas.92.12.5496. 7777537 PMC41722

[40] Bartolini A, Cardaci S, Lamba S, Oddo D, Marchiò C, Cassoni P, Amoreo CA, Corti G, Testori A, Bussolino F, Pasqualini R, Arap W, Corà D, et al. BCAM and LAMA5 Mediate the Recognition between Tumor Cells and the Endothelium in the Metastatic Spreading of KRAS-Mutant Colorectal Cancer. Clin Cancer Res. 2016; 22:4923–33. 10.1158/1078-0432.CCR-15-2664. 27143691

[41] Moulder R, Goo YA, Goodlett DR. Label-Free Quantitation for Clinical Proteomics. Methods Mol Biol. 2016; 1410:65–76. 10.1007/978-1-4939-3524-6_4. 26867738

[42] Waring MJ, Arrowsmith J, Leach AR, Leeson PD, Mandrell S, Owen RM, Pairaudeau G, Pennie WD, Pickett SD, Wang J, Wallace O, Weir A. An analysis of the attrition of drug candidates from four major pharmaceutical companies. Nat Rev Drug Discov. 2015; 14:475–86. 10.1038/nrd4609. 26091267

[43] Hinshaw DC, Shevde LA. The Tumor Microenvironment Innately Modulates Cancer Progression. Cancer Res. 2019; 79:4557–66. 10.1158/0008-5472.CAN-18-3962. 31350295 PMC6744958

[44] Rugg-Gunn PJ, Cox BJ, Lanner F, Sharma P, Ignatchenko V, McDonald AC, Garner J, Gramolini AO, Rossant J, Kislinger T. Cell-surface proteomics identifies lineage-specific markers of embryo-derived stem cells. Dev Cell. 2012; 22:887–901. 10.1016/j.devcel.2012.01.005. 22424930 PMC3405530

[45] Stockwin LH, Blonder J, Bumke MA, Lucas DA, Chan KC, Conrads TP, Issaq HJ, Veenstra TD, Newton DL, Rybak SM. Proteomic analysis of plasma membrane from hypoxia-adapted malignant melanoma. J Proteome Res. 2006; 5:2996–3007. 10.1021/pr0601739. 17081051

[46] Dauphinee SM, Clayton A, Hussainkhel A, Yang C, Park YJ, Fuller ME, Blonder J, Veenstra TD, Karsan A. SASH1 is a scaffold molecule in endothelial TLR4 signaling. J Immunol. 2013; 191:892–901. 10.4049/jimmunol.1200583. 23776175

[47] Martinko AJ, Truillet C, Julien O, Diaz JE, Horlbeck MA, Whiteley G, Blonder J, Weissman JS, Bandyopadhyay S, Evans MJ, Wells JA. Targeting RAS-driven human cancer cells with antibodies to upregulated and essential cell-surface proteins. Elife. 2018; 7:e31098. 10.7554/eLife.31098. 29359686 PMC5796798

[48] Deberardinis RJ, Sayed N, Ditsworth D, Thompson CB. Brick by brick: metabolism and tumor cell growth. Curr Opin Genet Dev. 2008; 18:54–61. 10.1016/j.gde.2008.02.003. 18387799 PMC2476215

[49] Moreno-Sánchez R, Rodríguez-Enríquez S, Marín-Hernández A, Saavedra E. Energy metabolism in tumor cells. FEBS J. 2007; 274:1393–418. 10.1111/j.1742-4658.2007.05686.x. 17302740

[50] Warburg O. On the origin of cancer cells. Science. 1956; 123:309–14. 10.1126/science.123.3191.309. 13298683

[51] Semenza GL. HIF-1: mediator of physiological and pathophysiological responses to hypoxia. J Appl Physiol (1985). 2000; 88:1474–80. 10.1152/jappl.2000.88.4.1474. 10749844

[52] Imamura Y, Mukohara T, Shimono Y, Funakoshi Y, Chayahara N, Toyoda M, Kiyota N, Takao S, Kono S, Nakatsura T, Minami H. Comparison of 2D- and 3D-culture models as drug-testing platforms in breast cancer. Oncol Rep. 2015; 33:1837–43. 10.3892/or.2015.3767. 25634491

[53] Bhattacharya S, Calar K, de la Puente P. Mimicking tumor hypoxia and tumor-immune interactions employing three-dimensional *in vitro* models. J Exp Clin Cancer Res. 2020; 39:75. 10.1186/s13046-020-01583-1. 32357910 PMC7195738

[54] Beloribi-Djefaflia S, Vasseur S, Guillaumond F. Lipid metabolic reprogramming in cancer cells. Oncogenesis. 2016; 5:e189. 10.1038/oncsis.2015.49. 26807644 PMC4728678

[55] Seguin F, Carvalho MA, Bastos DC, Agostini M, Zecchin KG, Alvarez-Flores MP, Chudzinski-Tavassi AM, Coletta RD, Graner E. The fatty acid synthase inhibitor orlistat reduces experimental metastases and angiogenesis in B16-F10 melanomas. Br J Cancer. 2012; 107:977–87. 10.1038/bjc.2012.355. 22892389 PMC3464771

[56] Baenke F, Peck B, Miess H, Schulze A. Hooked on fat: the role of lipid synthesis in cancer metabolism and tumour development. Dis Model Mech. 2013; 6:1353–63. 10.1242/dmm.011338. 24203995 PMC3820259

[57] Zaidi N, Lupien L, Kuemmerle NB, Kinlaw WB, Swinnen JV, Smans K. Lipogenesis and lipolysis: the pathways exploited by the cancer cells to acquire fatty acids. Prog Lipid Res. 2013; 52:585–89. 10.1016/j.plipres.2013.08.005. 24001676 PMC4002264

[58] Maniotis AJ, Folberg R, Hess A, Seftor EA, Gardner LM, Pe’er J, Trent JM, Meltzer PS, Hendrix MJ. Vascular channel formation by human melanoma cells *in vivo* and *in vitro*: vasculogenic mimicry. Am J Pathol. 1999; 155:739–52. 10.1016/S0002-9440(10)65173-5. 10487832 PMC1866899

[59] Hendrix MJ, Seftor EA, Seftor RE, Chao JT, Chien DS, Chu YW. Tumor cell vascular mimicry: Novel targeting opportunity in melanoma. Pharmacol Ther. 2016; 159:83–92. 10.1016/j.pharmthera.2016.01.006. 26808163 PMC4779708

[60] Rezaeeyan H, Shahrabi S, McKee TD, Saki N. The expression of CD markers in solid tumors: Significance in metastasis and prognostic value. Histol Histopathol. 2018; 33:1005–12. 10.14670/HH-11-981. 29508889

[61] Krishnamurti U, Silverman JF. HER2 in breast cancer: a review and update. Adv Anat Pathol. 2014; 21:100–07. 10.1097/PAP.0000000000000015. 24508693

[62] Fry TJ, Shah NN, Orentas RJ, Stetler-Stevenson M, Yuan CM, Ramakrishna S, Wolters P, Martin S, Delbrook C, Yates B, Shalabi H, Fountaine TJ, Shern JF, et al. CD22-targeted CAR T cells induce remission in B-ALL that is naive or resistant to CD19-targeted CAR immunotherapy. Nat Med. 2018; 24:20–28. 10.1038/nm.4441. 29155426 PMC5774642

[63] Sawyers CL. Herceptin: A First Assault on Oncogenes that Launched a Revolution. Cell. 2019; 179:8–12. 10.1016/j.cell.2019.08.027. 31519311

[64] Moroz A, Wang YH, Sharib JM, Wei J, Zhao N, Huang Y, Chen Z, Martinko AJ, Zhuo J, Lim SA, Zhang LH, Seo Y, Carlin S, et al. Theranostic Targeting of CUB Domain Containing Protein 1 (CDCP1) in Pancreatic Cancer. Clin Cancer Res. 2020; 26:3608–15. 10.1158/1078-0432.CCR-20-0268. 32341034 PMC7367754

[65] Misale S, Fatherree JP, Cortez E, Li C, Bilton S, Timonina D, Myers DT, Lee D, Gomez-Caraballo M, Greenberg M, Nangia V, Greninger P, Egan RK, et al. KRAS G12C NSCLC Models Are Sensitive to Direct Targeting of KRAS in Combination with PI3K Inhibition. Clin Cancer Res. 2019; 25:796–807. 10.1158/1078-0432.CCR-18-0368. 30327306

[66] Stalin J, Nollet M, Garigue P, Fernandez S, Vivancos L, Essaadi A, Muller A, Bachelier R, Foucault-Bertaud A, Fugazza L, Leroyer AS, Bardin N, Guillet B, et al. Targeting soluble CD146 with a neutralizing antibody inhibits vascularization, growth and survival of CD146-positive tumors. Oncogene. 2016; 35:5489–500. 10.1038/onc.2016.83. 27065325

[67] Edlund K, Lindskog C, Saito A, Berglund A, Pontén F, Göransson-Kultima H, Isaksson A, Jirström K, Planck M, Johansson L, Lambe M, Holmberg L, Nyberg F, et al. CD99 is a novel prognostic stromal marker in non-small cell lung cancer. Int J Cancer. 2012; 131:2264–73. 10.1002/ijc.27518. 22392539

[68] Liu Y, Beyer A, Aebersold R. On the Dependency of Cellular Protein Levels on mRNA Abundance. Cell. 2016; 165:535–50. 10.1016/j.cell.2016.03.014. 27104977

[69] Lundberg E, Fagerberg L, Klevebring D, Matic I, Geiger T, Cox J, Algenäs C, Lundeberg J, Mann M, Uhlen M. Defining the transcriptome and proteome in three functionally different human cell lines. Mol Syst Biol. 2010; 6:450. 10.1038/msb.2010.106. 21179022 PMC3018165

[70] Zhang X, Wang Z, Kang Y, Li X, Ma X, Ma L. MCAM expression is associated with poor prognosis in non-small cell lung cancer. Clin Transl Oncol. 2014; 16:178–83. 10.1007/s12094-013-1057-6. 23749325

[71] Gentles AJ, Bratman SV, Lee LJ, Harris JP, Feng W, Nair RV, Shultz DB, Nair VS, Hoang CD, West RB, Plevritis SK, Alizadeh AA, Diehn M. Integrating Tumor and Stromal Gene Expression Signatures With Clinical Indices for Survival Stratification of Early-Stage Non-Small Cell Lung Cancer. J Natl Cancer Inst. 2015; 107:djv211. 10.1093/jnci/djv211. 26286589 PMC6090873

[72] Ghimessy A, Radeczky P, Laszlo V, Hegedus B, Renyi-Vamos F, Fillinger J, Klepetko W, Lang C, Dome B, Megyesfalvi Z. Current therapy of KRAS-mutant lung cancer. Cancer Metastasis Rev. 2020; 39:1159–77. 10.1007/s10555-020-09903-9. 32548736 PMC7680319

[73] Santana-Codina N, Chandhoke AS, Yu Q, Małachowska B, Kuljanin M, Gikandi A, Stańczak M, Gableske S, Jedrychowski MP, Scott DA, Aguirre AJ, Fendler W, Gray NS, Mancias JD. Defining and Targeting Adaptations to Oncogenic KRAS^G12C^ Inhibition Using Quantitative Temporal Proteomics. Cell Rep. 2020; 30:4584–99.e4. 10.1016/j.celrep.2020.03.021. 32234489

[74] Gildea JJ, McGrath HE, Van Sciver RE, Wang DB, Felder RA. Isolation, growth, and characterization of human renal epithelial cells using traditional and 3D methods. Methods Mol Biol. 2013; 945:329–45. 10.1007/978-1-62703-125-7_20. 23097116

[75] Käll L, Canterbury JD, Weston J, Noble WS, MacCoss MJ. Semi-supervised learning for peptide identification from shotgun proteomics datasets. Nat Methods. 2007; 4:923–25. 10.1038/nmeth1113. 17952086

[76] Carvalho PC, Hewel J, Barbosa VC, Yates JR 3rd. Identifying differences in protein expression levels by spectral counting and feature selection. Genet Mol Res. 2008; 7:342–56. 10.4238/vol7-2gmr426. 18551400 PMC2703009

[77] Benjamini Y, Hochberg Y. Controlling the False Discovery Rate: a Practical and Powerful Approach to Multiple Testing. J Roy Stat Soc B Met. 1995; 57:289–300. 10.1111/j.2517-6161.1995.tb02031.x.

[78] Mi H, Muruganujan A, Casagrande JT, Thomas PD. Large-scale gene function analysis with the PANTHER classification system. Nat Protoc. 2013; 8:1551–66. 10.1038/nprot.2013.092. 23868073 PMC6519453

[79] Ennen M, Keime C, Gambi G, Kieny A, Coassolo S, Thibault-Carpentier C, Margerin-Schaller F, Davidson G, Vagne C, Lipsker D, Davidson I. *MITF*-High and *MITF*-Low Cells and a Novel Subpopulation Expressing Genes of Both Cell States Contribute to Intra- and Intertumoral Heterogeneity of Primary Melanoma . Clin Cancer Res. 2017; 23:7097–107. 10.1158/1078-0432.CCR-17-0010. 28855355

