# Supplementary material for: Comparative microsomal proteomics of a model lung cancer cell line NCI-H23 reveals distinct differences between molecular profiles of 3D and 2D cultured cells
Source: Oncotarget. 2021 Sep 28;12(20):2022–38. doi: 10.18632/oncotarget.28072 (PMC8487723; doi:10.18632/oncotarget.28072)
Supplement: Supplementary file 1 [file oncotarget-12-2022-s001.pdf]

Comparative microsomal proteomics of a model lung cancer cell line NCI-H23 reveals distinct differences between molecular profiles of 3D and 2D cultured cells

SUPPLEMENTARY MATERIALS

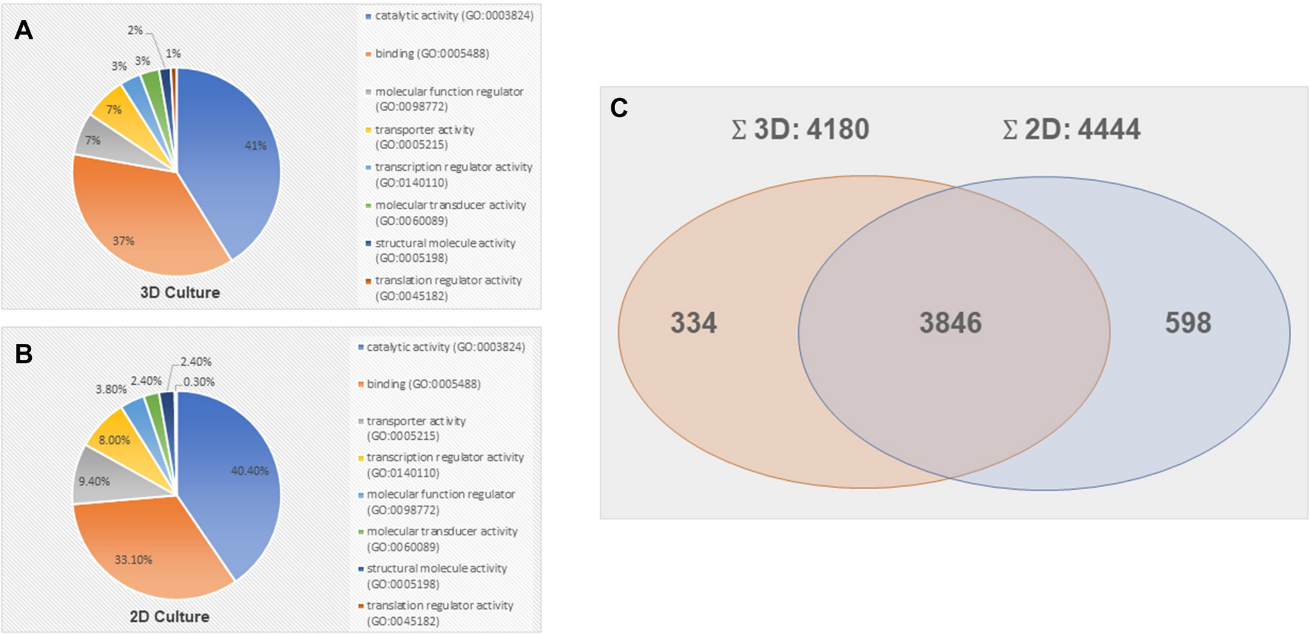

Supplementary Figure 1: PANTHER analysis: distribution of molecular functions of differentially expressed proteins identified NCl-H23 cells grown in 3D culture (**A**) and 2D culture (**B**). Venn diagrams showing overlapping protein identifications in microsomal fraction obtained from NCl-H23 cells grown in 3D and 2D culture, respectively, corresponding to a total of 4,778 non-redundant protein identifications (**C**).

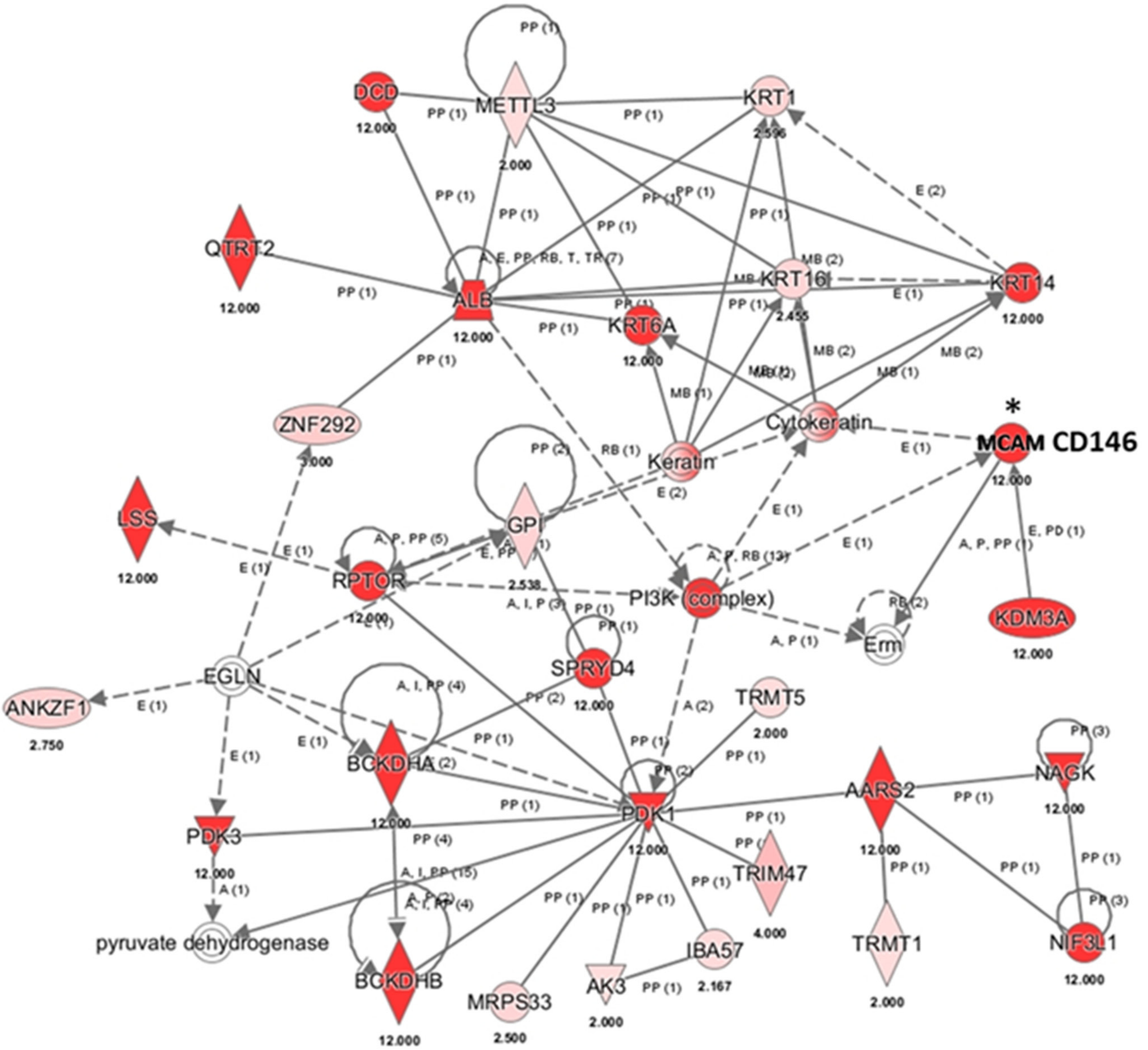

Supplementary Figure 2: IPA ® network analysis, 3D cultured cells, network 2, top functions: cancer, angiogenesis. Red color depicts proteins identified solely in 3D cultured cells, pink color depicts proteins. Solid lines show direct interactions, dotted lines show indirect protein-protein interactions described in the literature. Asterisk marks a CD molecule selected for cross-validation (i.e., CD146-MCAM).

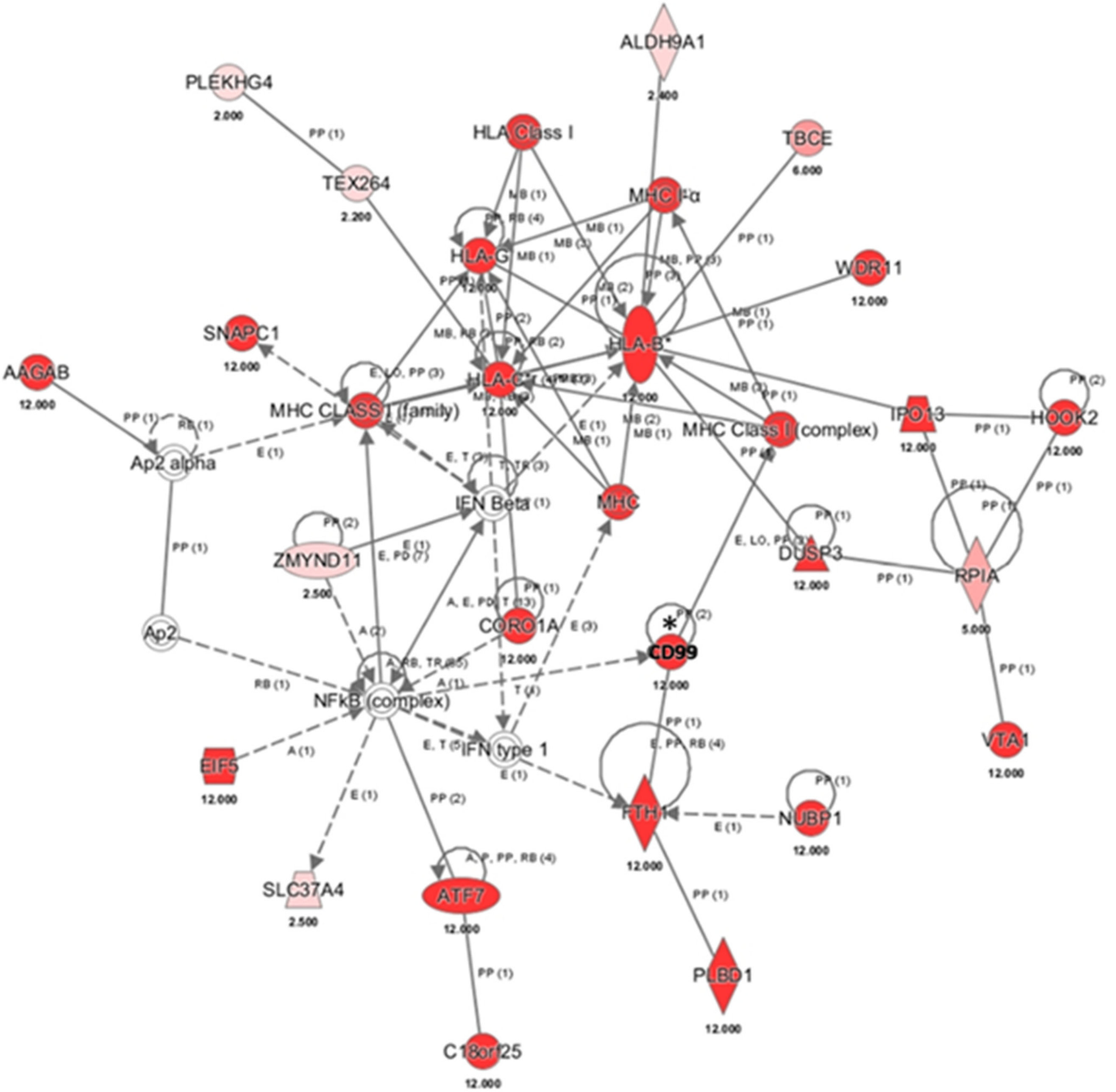

Supplementary Figure 3: IPA ® network analysis, 3D cultured cells, network 6, top functions: cellular growth, proliferation. Red color depicts proteins identified solely in 3D cultured cells, pink color depicts proteins. Solid lines show direct interactions, dotted lines show indirect protein-protein interactions described in the literature. Asterisk marks a molecule selected for cross-validation (i.e., CD99).

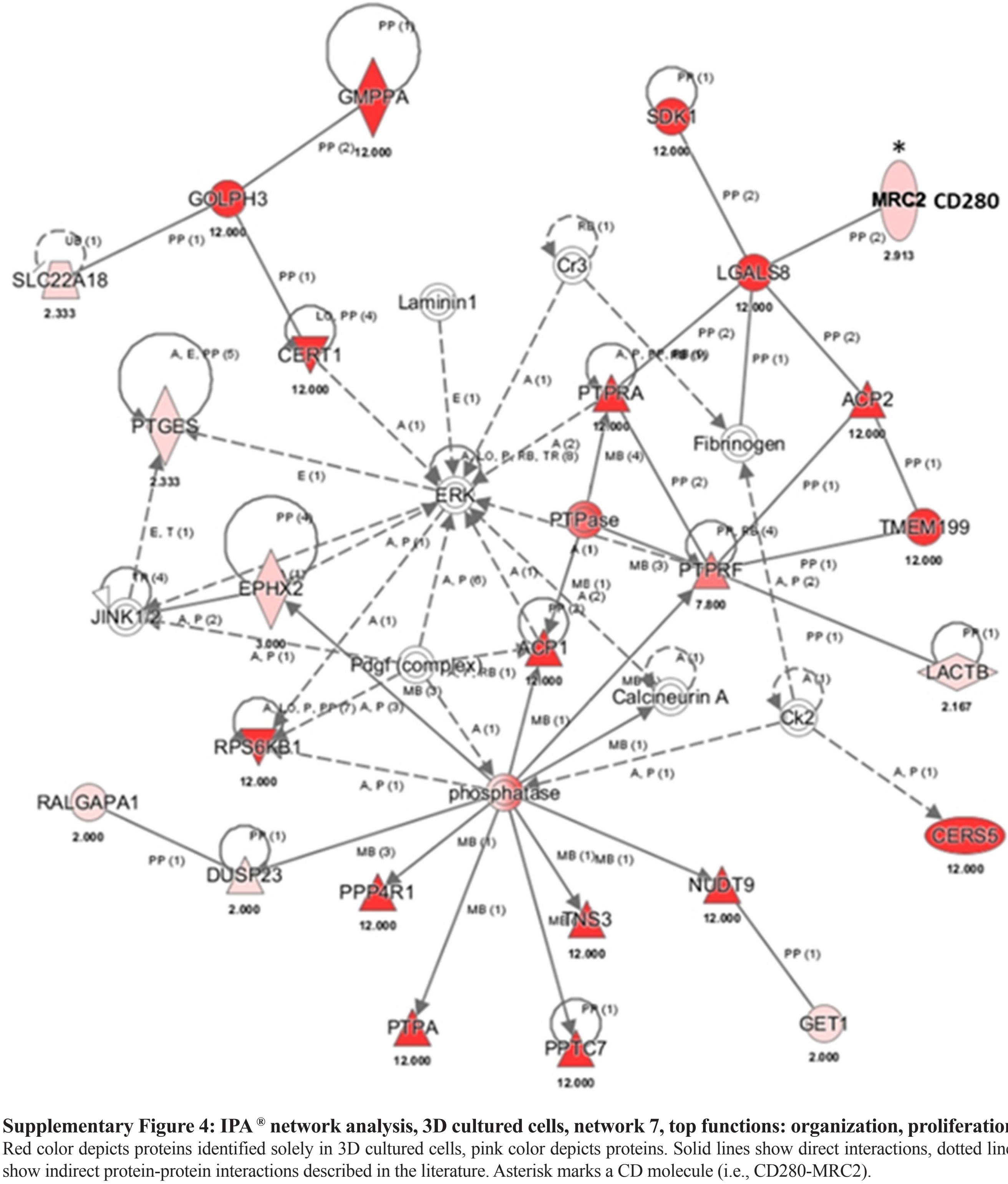

Supplementary Figure 4: IPA ® network analysis, 3D cultured cells, network 7, top functions: organization, proliferation. Red color depicts proteins identified solely in 3D cultured cells, pink color depicts proteins. Solid lines show direct interactions, dotted lines show indirect protein-protein interactions described in the literature. Asterisk marks a CD molecule (i.e., CD280-MRC2).

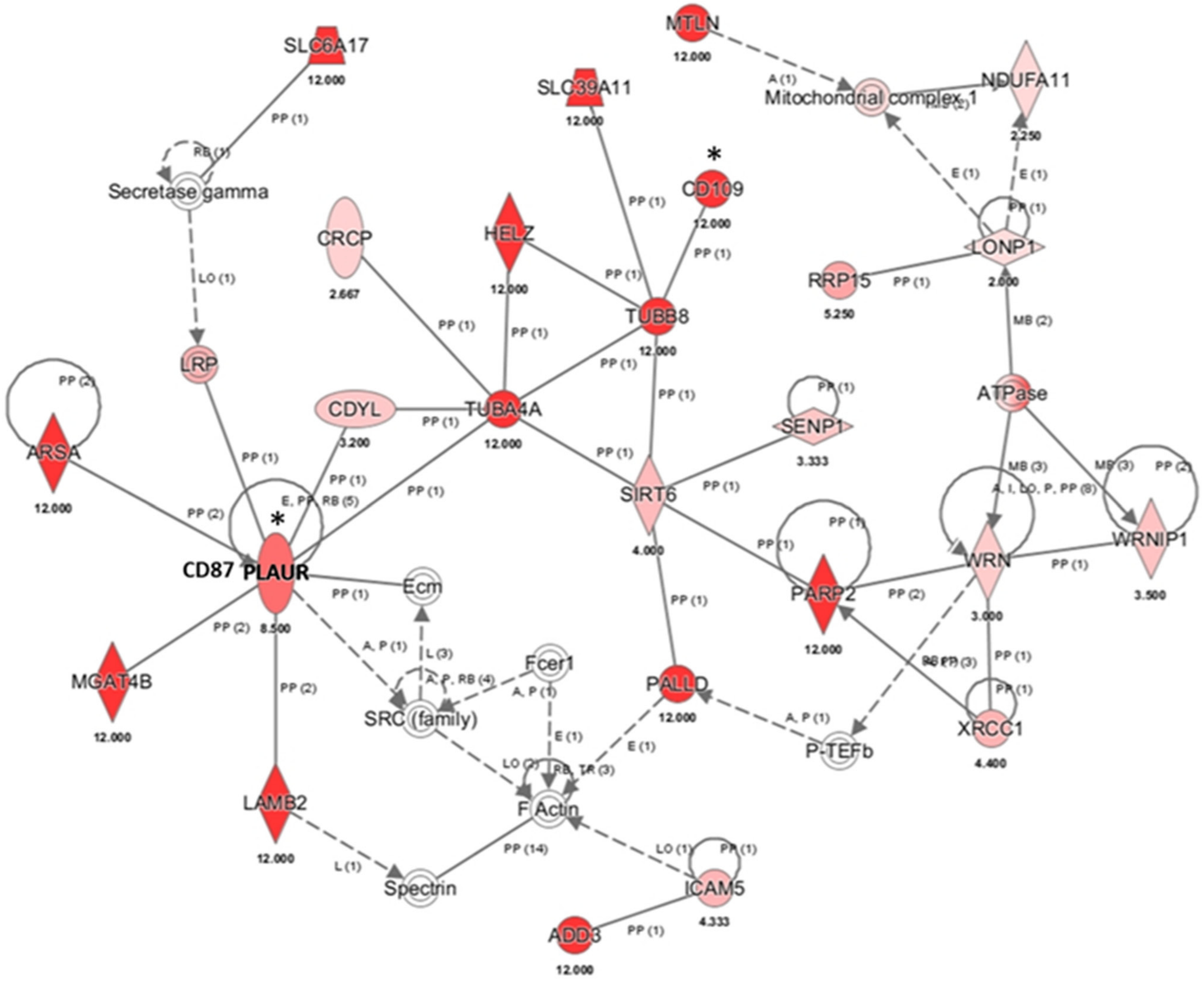

Supplementary Figure 5: IPA ® network analysis, 3D cultured cells, network 8, top function: cancer. Red color depicts proteins identified solely in 3D cultured cells, pink color depicts proteins. Solid lines show direct interactions, dotted lines show indirect protein-protein interactions described in the literature. Asterisk marks a CD molecule (i.e., CD87-PLAUR, CD109).

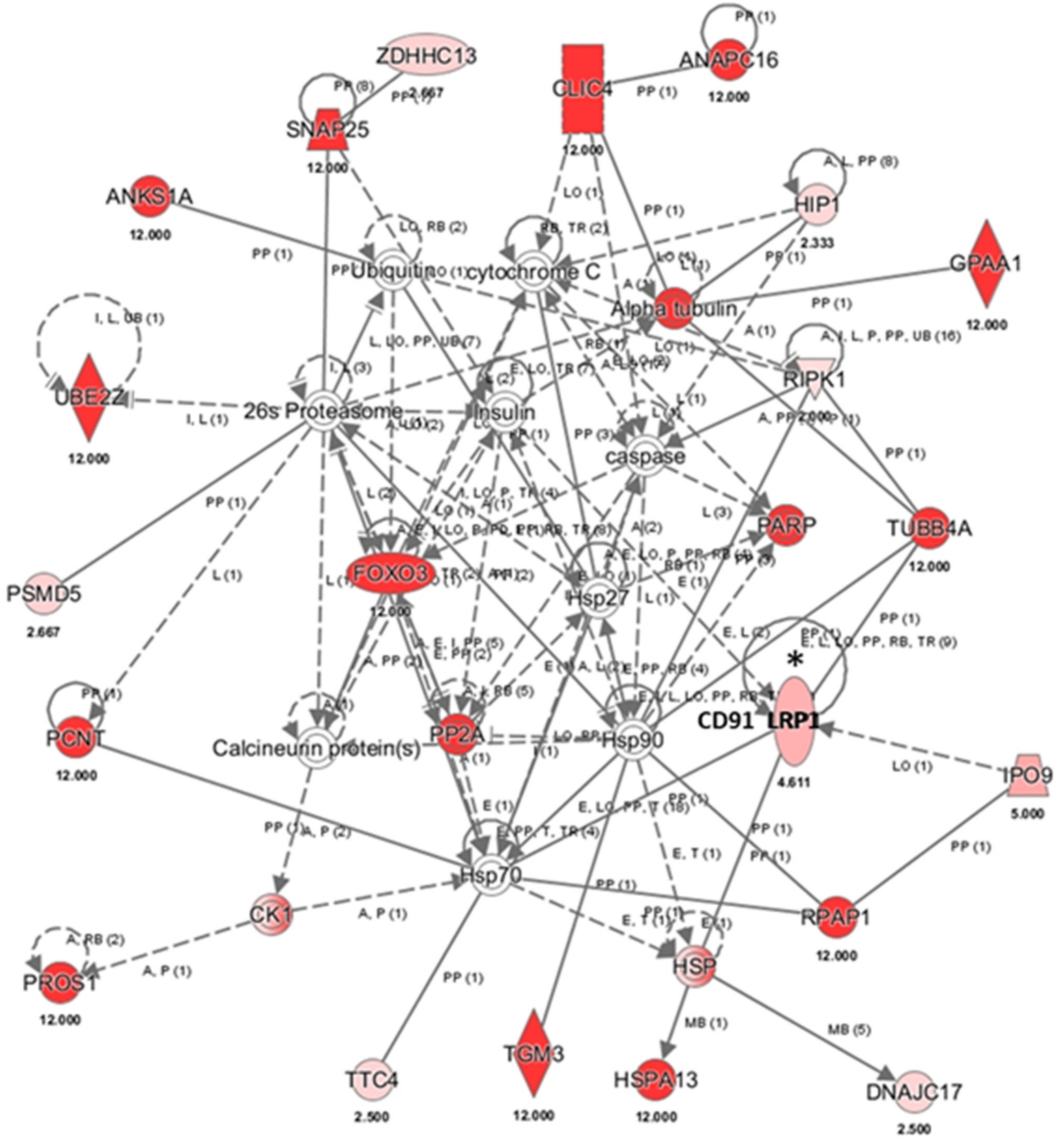

Supplementary Figure 6: IPA® network analysis, 3D cultured cells, network 10, top functions: organization, angiogenesis. Red color depicts proteins identified solely in 3D cultured cells, pink color depicts proteins. Solid lines show direct interactions, dotted lines show indirect protein-protein interactions described in the literature. Asterisk marks a CD molecule selected for cross-validation (i.e.,CD91-LRP 1).

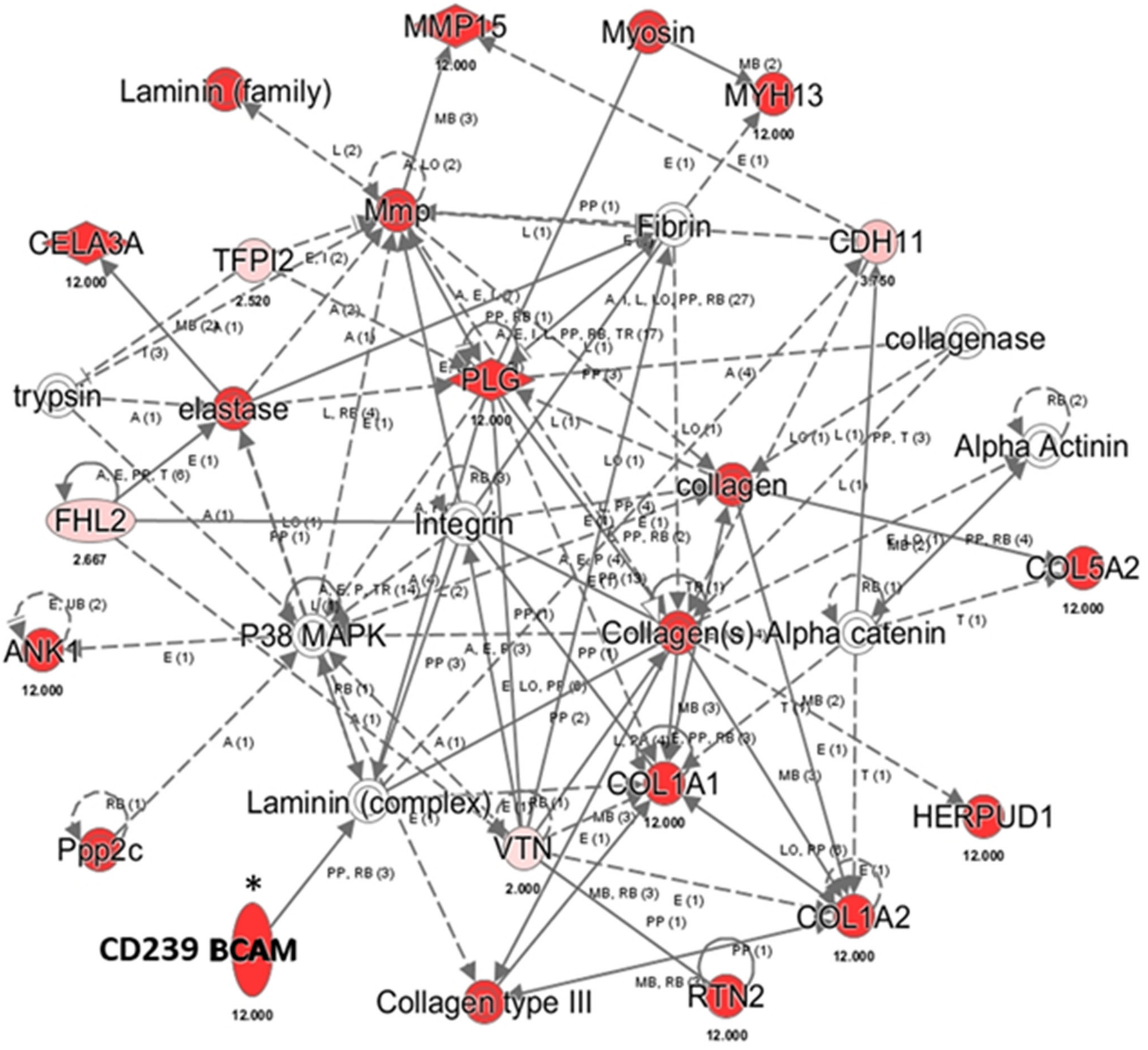

Supplementary Figure 7: IPA ® network analysis, 3D cultured cells, network 17, top functions: connective tissue disorder, angiogenesis. Red color depicts proteins identified solely in 3D cultured cells, pink color depicts proteins. Solid lines show direct interactions, dotted lines show indirect protein-protein interactions described in the literature. Asterisk marks a CD molecule selected for cross-validation (i.e., CD239-BCAM).

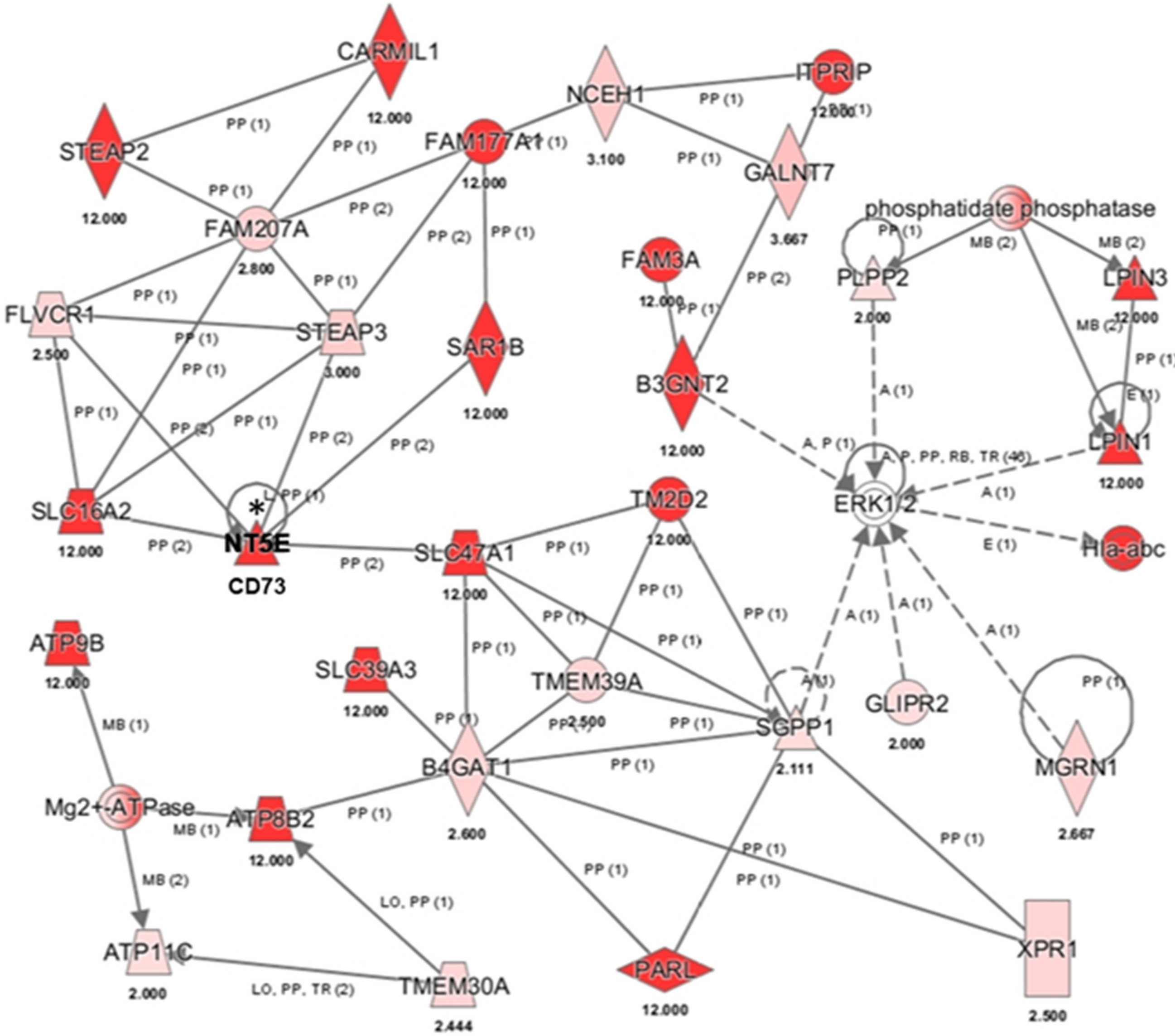

Supplementary Figure 8: IPA ® network analysis, 2D cultured cells, network 4, top functions: cancer, metastasis. Red color depicts proteins identified solely in 3D cultured cells, pink color depicts proteins. Solid lines show direct interactions, dotted lines show indirect protein-protein interactions described in the literature. Asterisk marks a CD molecule (i.e., CD73).

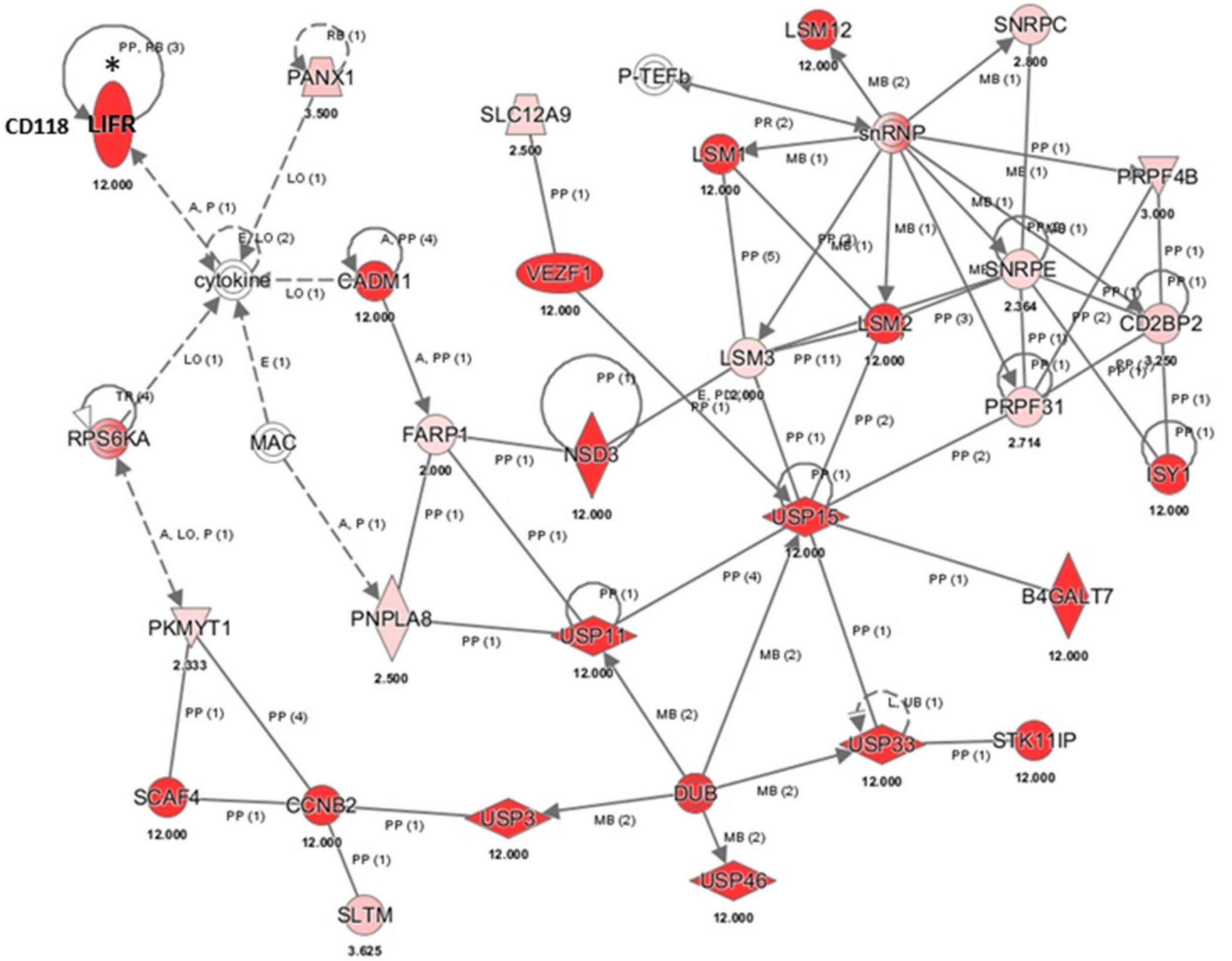

Supplementary Figure 9: IPA ® network analysis, 2D cultured cells, network 4, top functions: metastasis, chemotaxis. Red color depicts proteins identified solely in 3D cultured cells, pink color depicts proteins. Solid lines show direct interactions, dotted lines show indirect protein-protein interactions described in the literature. Asterisk marks a CD molecule (i.e., CD118).

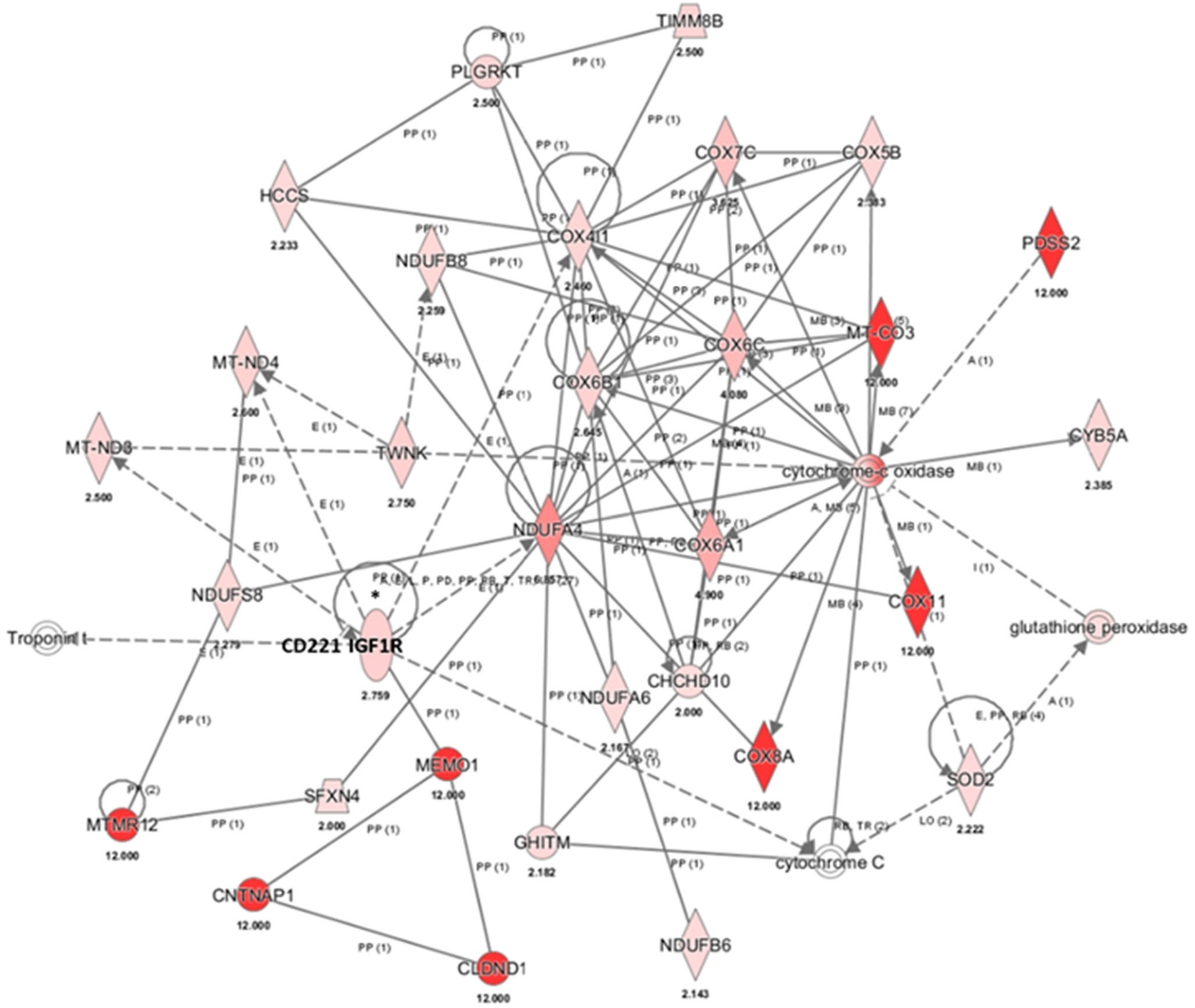

Supplementary Figure 10: IPA ® network analysis, 2D cultured cells, network 8, top function: developmental disorder. Red color depicts proteins identified solely in 3D cultured cells, pink color depicts proteins. Solid lines show direct interactions, dotted lines show indirect protein-protein interactions described in the literature. Asterisk marks a CD molecule (i.e., CD221-IGF1R).

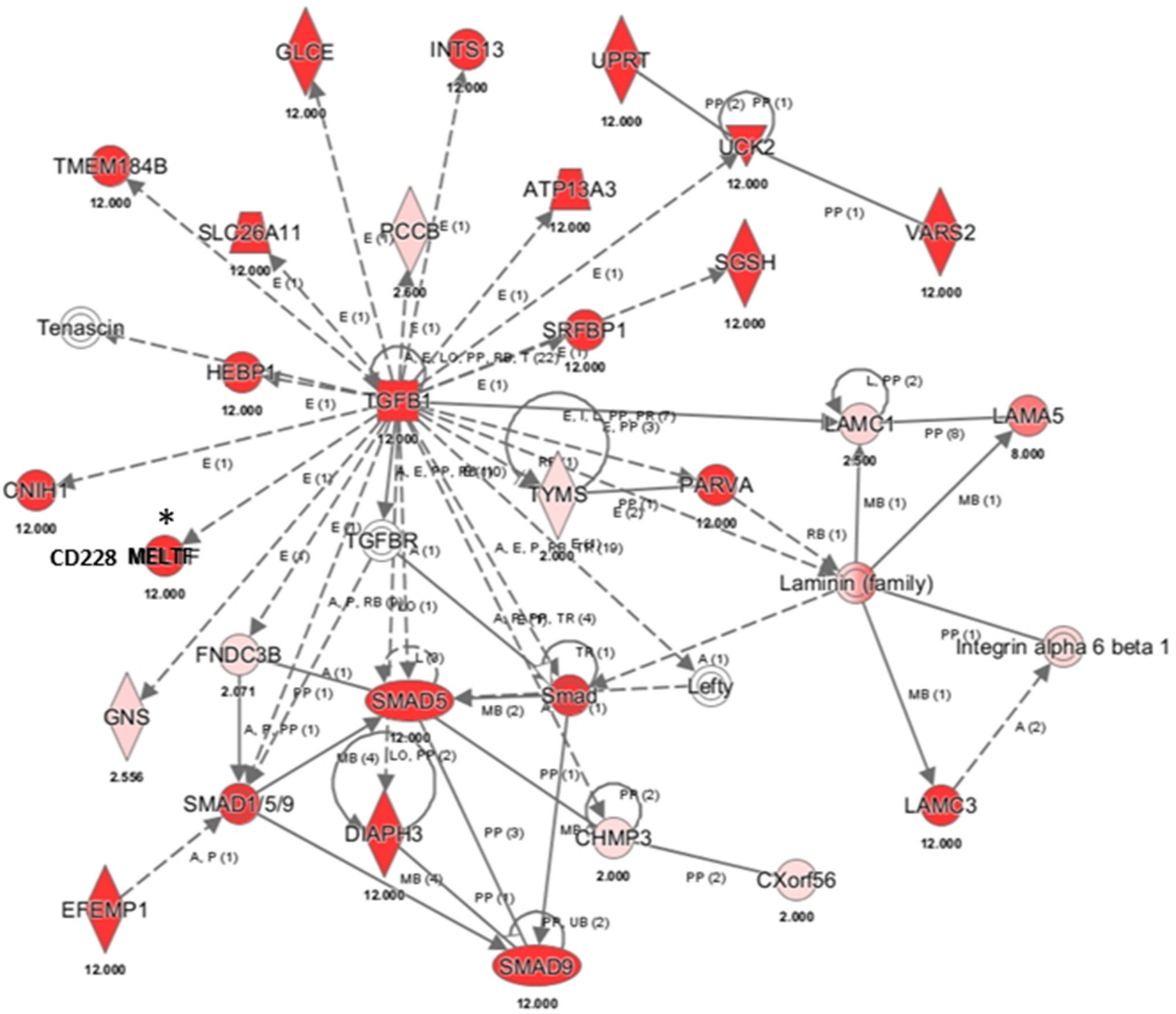

Supplementary Figure 11: IPA ® network analysis, 2D cultured cells, network 14, top function: cellular growth. Red color depicts proteins identified solely in 3D cultured cells, pink color depicts proteins. Solid lines show direct interactions, dotted lines show indirect protein-protein interactions described in the literature. Asterisk marks a CD molecule (i.e., CD228).

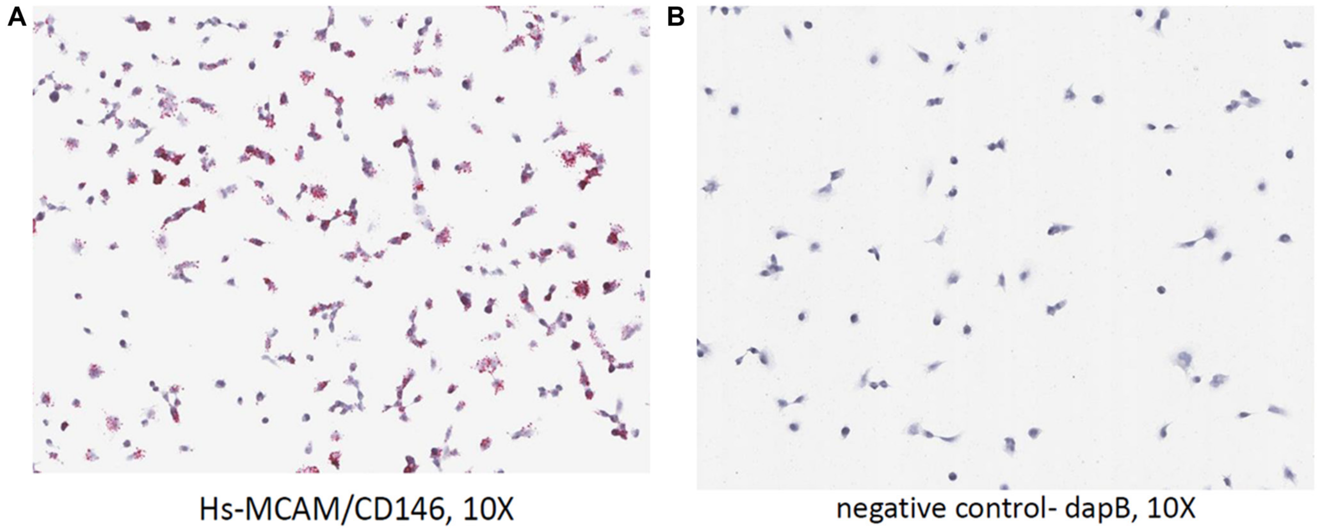

Supplementary Figure 12: CD146 Staining for Human HT23 cultured cells with RNAscope 2.5 HD red assay. Image of 10X show MCAM/CD146 probe detecting low to moderate RNA staining in most of the cultured cells (**A**). Representative image of 10X show negative control (**B**).

A

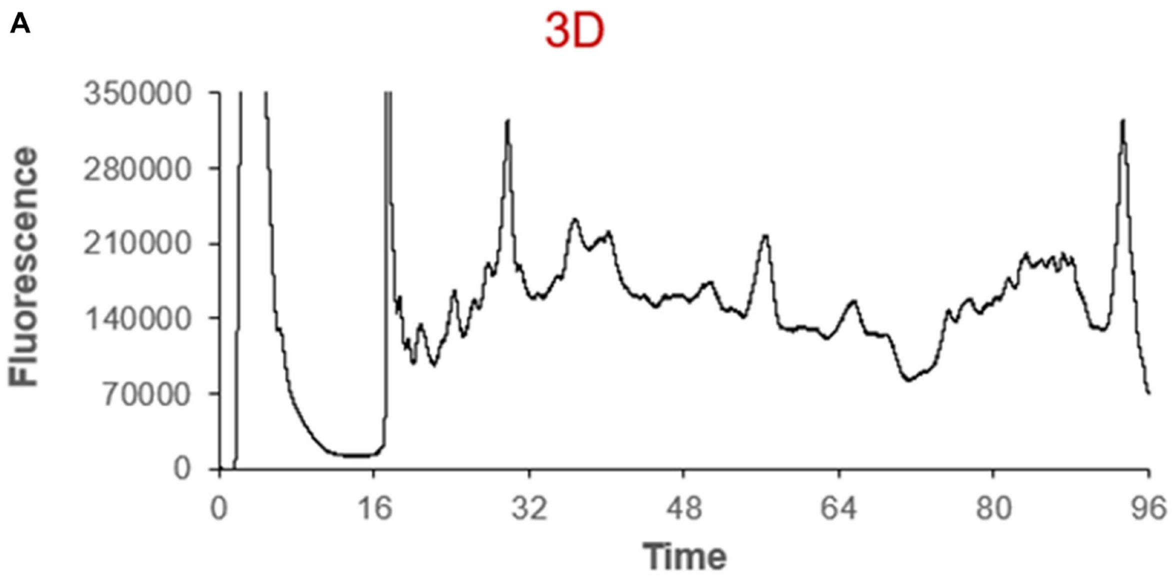

B

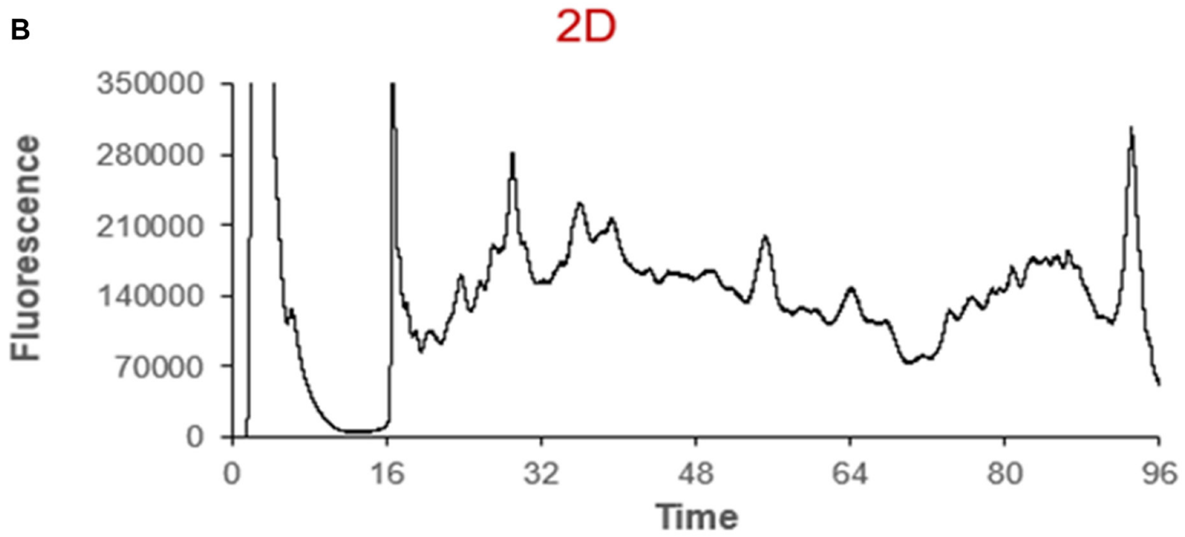

Supplementary Figure 13: SCX fractionation. (**A**) SCX chromatogram depicting separation of peptides generated from 3D-cultured NCl-H23 cells. (**B**) SCX chromatogram depicting separation of peptides generated from 2D-cultured NCl-H23 cells.

Supplementary Table 1: Proteins groups identified in membrane fraction of 2D-cultured NCI-H23 cells, showing subcellular location data for a given protein group currently available in human proteom atlas (HPA). See Supplementary Table 1

Supplementary Table 2: Proteins groups identified in membrane fraction of 2D-cultured NCI-H23 cells, showing subcellular location data for a given protein group currently available in human proteom atlas (HPA). See Supplementary Table 2

Supplementary Table 3: Peptide groups identified in membrane fraction of 3D-cultured NCI-H23 cells. See Supplementary Table 3

Supplementary Table 4: Peptide groups identified in membrane fraction of 2D-cultured NCI-H23 cells. See Supplementary Table 4

Supplementary Table 5: Integral membrane proteins identified in 3D-cultured NCI-H23 cells. See Supplementary Table 5

Supplementary Table 6: Integral membrane proteins identified in 2D-cultured NCI-H23 cells. See Supplementary Table 6

Supplementary Table 7: Cell surface proteins, including CD molecules, identified in 3D-cultured NCI-H23 cells. See Supplementary Table 7

Supplementary Table 8: Cell surface proteins, including CD molecules, identified in 2D-cultured NCI-H23 cells. See Supplementary Table 8

Supplementary Table 9: Protein groups identified in both 3D- and 2D-cultured NCI-H23 cells. See Supplementary Table 9

Supplementary Table 10: Protein groups identified exclusively in 3D-cultured NCI-H23 cells. See Supplementary Table 10

Supplementary Table 11: Protein groups identified exclusively in 2D-cultured NCI-H23 cells. See Supplementary Table 11

Supplementary Table 12: Proteins regulated in culture-dependent manmer in 3D and 2D cultured NCI-H23 cells using shotgun microsomal proteomics. See Supplementary Table 12

Supplementary Table 13: Cell surface proteins identified exclusively in 3D-cultured NCI-H23 cells

Accession	Description	Gene	# PSMs	CD	GPI
Q9UQ53	Alpha-1,3-mannosyl-glycoprotein 4-beta-N-acetylglucosaminyltransferase B OS	MGAT4B	2	no	0
P02765	Alpha-2-HS-glycoprotein OS	AHSG	15	no	0
O95477	ATP-binding cassette sub-family A member 1 OS	ABCA1	3	no	0
P50895	Basal cell adhesion molecule OS	BCAM	26	CD239	0
Q6YHK3	CD109 antigen OS	CD109	21	CD109	1
P14209	CD99 antigen OS	CD99	22	CD99	0
P43121	Cell surface glycoprotein MUC18 OS	MCAM	72	CD146	0
P02452	Collagen alpha-1(I) chain OS	COL1A1	30	no	0
P08123	Collagen alpha-2(I) chain OS	COL1A2	17	no	0
P05997	Collagen alpha-2(V) chain OS	COL5A2	2	no	0
Q9UJ14	Gamma-glutamyltransferase 7 OS	GGT7	3	no	0
P48723	Heat shock 70 kDa protein 13 OS	HSPA13	2	no	0
Q68CP4	Heparan-alpha-glucosaminide N-acetyltransferase OS	HGSNAT	2	no	0
Q9Y287	Integral membrane protein 2B OS	ITM2B	2	no	0
P78552	Interleukin-13 receptor subunit alpha-1 OS	IL13RA1	2	CD213a1	0
Q7Z4H8	KDEL motif-containing protein 2 OS	KDELC2	3	no	0
P55268	Laminin subunit beta-2 OS	LAMB2	3	no	0
P11117	Lysosomal acid phosphatase OS	ACP2	4	no	0
Q9UJJ9	N-acetylglucosamine-1-phosphotransferase subunit gamma OS	GNPTG	2	no	0
Q8N2Q7	Neuroligin-1 OS	NLGN1	3	no	0
Q6P4A8	Phospholipase B-like 1 OS	PLBD1	4	no	0
Q7Z5N4	Protein sidekick-1 OS	SDK1	2	no	0
Q8NHP8	Putative phospholipase B-like 2 OS	PLBD2	6	no	0
P18433	Receptor-type tyrosine-protein phosphatase alpha OS	PTPRA	5	no	0
O75094	Slit homolog 3 protein OS	SLIT3	5	no	0
P53794	Sodium/myo-inositol cotransporter OS	SLC5A3	2	no	0
Q9UGH3	Solute carrier family 23 member 2 OS	SLC23A2	3	no	0
P10646	Tissue factor pathway inhibitor OS	TFPI	2	no	0
P68366	Tubulin alpha-4A chain OS	TUBA4A	620	no	0
P04350	Tubulin beta-4A chain OS	TUBB4A	1623	no	0
Q13433	Zinc transporter ZIP6 OS	SLC39A6	3	no	0

Supplementary Table 14: Cell surface proteins identified exclusively in 2D-cultured NCI-H23 cells

Accession #	Description	Gene	# PSMs	CD	GPI
P21589	5′-nucleotidase OS	NT5E	8	CD73	1
Q92485	Acid sphingomyelinase-like phosphodiesterase 3b OS	SMPDL3B	2	no	0
O43306	Adenylate cyclase type 6 OS	ADCY6	3	no	0
P06280	Alpha-galactosidase A OS	GLA	2	no	0
A1A5B4	Anoctamin-9 OS	ANO9	3	no	0
Q9H6X2	Anthrax toxin receptor 1 OS	ANTXR1	6	no	0
Q9NR09	Baculoviral IAP repeat-containing protein 6 OS	BIRC6	3	no	0
P98160	Basement membrane-specific heparan sulfate proteoglycan core protein OS	HSPG2	2	no	0
Q13286	Battenin OS	CLN3	3	no	0
Q9BY67	Cell adhesion molecule 1 OS	CADM1	7	no	0
Q9P2E5	Chondroitin sulfate glucuronyltransferase OS	CHPF2	2	no	0
Q9NY35	Claudin domain-containing protein 1 OS	CLDND1	3	no	0
P78357	Contactin-associated protein 1 OS	CNTNAP1	6	no	0
O94923	D-glucuronyl C5-epimerase OS	GLCE	2	no	0
Q12805	EGF-containing fibulin-like extracellular matrix protein 1 OS	EFEMP1	4	no	0
Q9GZR5	Elongation of very long chain fatty acids protein 4 OS	ELOVL4	3	no	0
P29323	Ephrin type-B receptor 2 OS	EPHB2	18	no	0
P41440	Folate transporter 1 OS	SLC19A1	18	no	0
P05534	HLA class I histocompatibility antigen, A-24 alpha chain OS	HLA-A	250	no	0
P01889	HLA class I histocompatibility antigen, B-7 alpha chain OS	HLA-B	151	no	0
P04222	HLA class I histocompatibility antigen, Cw-3 alpha chain OS	HLA-C	137	no	0
P10321	HLA class I histocompatibility antigen, Cw-7 alpha chain OS	HLA-C	245	no	0
Q12891	Hyaluronidase-2 OS	HYAL2	2	no	1
Q8IWB1	Inositol 1,4,5-trisphosphate receptor-interacting protein OS	ITPRIP	14	no	0
Q6UWB1	Interleukin-27 receptor subunit alpha OS	IL27RA	2	no	0
P42702	Leukemia inhibitory factor receptor OS	LIFR	2	CD118	0
Q7Z4F1	Low-density lipoprotein receptor-related protein 10 OS	LRP10	7	no	0
P08582	Melanotransferrin OS	MELTF	5	CD228	1
Q9UNW1	Multiple inositol polyphosphate phosphatase 1 OS	MINPP1	8	no	0
P34059	N-acetylgalactosamine-6-sulfatase OS	GALNS	2	no	0
Q9UK23	N-acetylglucosamine-1-phosphodiester alpha-N-acetylglucosaminidase OS	NAGPA	5	no	0
Q92626	Peroxidasin homolog OS	PXDN	2	no	0
P53801	Pituitary tumor-transforming gene 1 protein-interacting protein OS	PTTG1IP	4	no	0
Q9Y4D7	Plexin-D1 OS	PLXND1	2	no	0
Q5SGD2	Protein phosphatase 1L OS	PPM1L	2	no	0
Q96NT5	Proton-coupled folate transporter OS	SLC46A1	2	no	0
Q15262	Receptor-type tyrosine-protein phosphatase kappa OS	PTPRK	2	no	0
Q8WTV0	Scavenger receptor class B member 1 OS	SCARB1	5	no	0
Q9H2H9	Sodium-coupled neutral amino acid transporter 1 OS	SLC38A1	2	no	0
Q9BXS9	Solute carrier family 26 member 6 OS	SLC26A6	6	no	0
Q12770	Sterol regulatory element-binding protein cleavage-activating protein OS	SCAP	5	no	0
P50443	Sulfate transporter OS	SLC26A2	9	no	0
Q8TB96	T-cell immunomodulatory protein OS	ITFG1	5	no	0
Q96AE7	Tetratricopeptide repeat protein 17 OS	TTC17	3	no	0
P01137	Transforming growth factor beta-1 OS	TGFB1	6	no	0
Q9BVX2	Transmembrane protein 106C OS	TMEM106C	13	no	0
Q9H813	Transmembrane protein 206 OS	TMEM206	3	no	0
Q8NBN3	Transmembrane protein 87A OS	TMEM87A	12	no	0
Q96K49	Transmembrane protein 87B OS	TMEM87B	6	no	0
Q9NY97	UDP-GlcNAc:betaGal beta-1,3-N-acetylglucosaminyltransferase 2 OS	B3GNT2	3	no	0

Supplementary Table 15A: Top biological function enriched in 3D cultured cells

CONNECTIVE TISSUE DEVELOPMENT

Functions Annotation	*p*-value	# Molecules	Molecules	CD Molecules
Cell proliferation of fibroblasts	1.19E-07	31	ACP1,ALB,ARHGEF11,ARID3A,CEBPB,CTC1,EZH2,FADD,FANCD2,FTH1,GPI,HDGFL2,HGS,HMOX1,KDM5A,KMT2C,KMT5B,LATS1,MEN1,MORC3,NDRG1,PHF14,PLAUR,PTGES,SIN3A,SIRT6,SMAD3,TGIF1,TSC2,VTN,WRN	CD87
Growth of connective tissue	2.71E-06	45	ACP1,ALB,ARHGEF11,ARID3A,BTK,CD109,CEBPB,COL1A1,CSNK1A1,CTC1,EZH2,F2RL1,FADD,FANCD2,FTH1,GOLPH3,GPI,HDGFL2,HGS,HMOX1,KDM2B,KDM5A,KMT2C,KMT5B,LATS1,MEN1,MFN2,MORC3,NDRG1,PDK1,PHF14,PLAUR,PTGES,PTPRF,RRM1,SDC4,SIN3A,SIRT6,SMAD3,SMARCA4,TGIF1,TRAF4,TSC2,VTN,WRN	CD87, CD109
Cell movement of fibroblast cell lines	4.31E-06	19	ACP1,ARHGEF11,CLIC4,FHL2,GIT1,GPI,IPO9,KDM2B,LGALS8,NCK1,PALLD,PLAUR,PLG,PODXL2,PTPRA,TRAF4,TSC2,VTN,WASF3	CD87, CD280
Proliferation of connective tissue cells	7.36E-06	41	ACP1,ALB,ARHGEF11,ARID3A,CD109,CEBPB,COL1A1,CTC1,EZH2,F2RL1,FADD,FANCD2,FTH1,GOLPH3,GPI,HDGFL2,HGS,HMOX1,KDM2B,KDM5A,KMT2C,KMT5B,LATS1,MEN1,MORC3,NDRG1,PHF14,PLAUR,PTGES,PTPRF,RRM1,SDC4,SIN3A,SIRT6,SMAD3,SMARCA4,TGIF1,TRAF4,TSC2,VTN,WRN	CD87, CD109
Migration of fibroblast cell lines	3.28E-05	14	ACP1,ARHGEF11,CLIC4,FHL2,KDM2B,NCK1,PALLD,PLAUR,PLG,PTPRA,TRAF4,TSC2,VTN,WASF3	CD87
Binding of fibroblast cell lines	8.12E-05	11	ACP1,CDH11,FHL2,IL13RA1,LGALS8,NCK1,PALLD,PLAUR,SDC4,TFPI,TMEM123	CD87
Cell movement of fibroblasts	9.56E-04	14	COL1A1,LRP1,MRC2,NCK1,PALLD,PLAUR,PTPRA,RAP2A,SDC4,SMAD3,SNX17,TNS3,TSC2,VTN	CD87, CD91
Migration of fibroblasts	2.02E-03	11	COL1A1,LRP1,MRC2,PALLD,PLAUR,PTPRA,RAP2A,SDC4,SNX17,TSC2,VTN	CD87, CD91, CD280
Function of brown adipose tissue	2.44E-03	3	CEBPB,KDM3A,RIPK1	None
Diferentiation of fibroblasts	2.79E-03	8	CEBPB,F2RL1,FOXO3,KDM5A, MCAM,PARP2,PLAUR,SMARCA4	CD146, CD87

© 2000–2020 QIAGEN. All rights reserved.

Supplementary Table 15B: Top biological function enriched in 2D cultured cells

ORGANISMAL SURVIVAL

Functions Annotation	*p*-value	# Molecules	Molecules	CD Molecules
Morbidity or mortality	3.99E-09	260	ABCB10,ABCC1,ABHD5,ABI1,ACADM,ACTL6A,ACTN4,ADGRG6,ADM,AFF4,AGL,AHI1,AKAP13,ALDH5A1,ANTXR1,ARHGEF12,ARRB1,ATF2,ATF6,ATF6B,ATN1,ATP6V0C,ATP7A,B4GALT1,BARD1,BECN1,BIRC5,BIRC6,BRCA1,CASK,CASZ1,CAV1,CBS/CBSL,CC2D1A,CCDC134,CCNA2,CCNB2,CDK2,CDK2AP1,CEBPD,CHMP5,CIT,CLCN7,CLN3,CNOT3,CNTNAP1,COPS8,CPOX,CSK,CTNNA2,CUX1,DAB2,DAXX,DDR1,DIAPH3,DMD,DPAGT1,DVL2,ECD,ECSIT,EEF1E1,EFEMP1,EHD3,EHD4,EIF4E2,ELOVL1,ELOVL4,EP400,EPHB2,ERBIN,ERCC1,ERCC6,ERCC6L,ERF,ERRFI1,EXTL3,FITM2,FLVCR1,FNDC3B,FOS,FOSL1,FTL,GAS2L3,GATA3,GBE1,GCLM,GFAP,GJA1,GJC1,GLCE,GNAZ,GNG5,GPR108,HEXB,HLA-A,HMGCL,HSBP1,HSD3B7,HSPG2,HYAL2,IGF1R,IL27RA,INO80,JUNB,KIF1A,KIF20A,KIF3A,KRT8,LAMC1,LIFR,LIMA1,LIMK1,LRP6,LRP8,LRRC8A,MADD,MAN2A1,MAP1B,MBNL1,MBTPS1,MCL1,MCM3AP,MED24,MELK,MKI67,MMACHC,MTA2,MTERF3,MTF2,MTHFD2,MYH10,MYH9,MYO18A,NCAPG2,NCAPH2,NCOR1,NDC80,NEBL,NF1,NF2,NFATC1,NFIX,NLE1,NOA1,NOP53,NOTCH2,NSUN4,NT5E,NUS1,OAT,PAFAH1B1,PAM,PANK1,PARL,PATZ1,PCYT1A,PDSS2,PEA15,PEX5,PFKM,PGP,PI4KA,PINX1,PISD,PITPNA,PKD2,PLCB1,PLCG1,PLIN2,PLXND1,PNPLA2,PNPLA8,POLB,POLG,POLR2A,POMGNT1,POMGNT2,PPP1R13L,PRKCD,PRMT1,PRPF31,PSMB4,PSMG1,PTBP2,PTK2,PTPN2,PTPN9,PXN,PYGO2,RAD54B,RAD54L,RAF1,RAI1,RB1,RBBP6,RBM38,RFK,RICTOR,RIF1,RPS6KB2,SCARB1,SCD,SHB,SLC16A2,SLC19A1,SLC19A3,SLC20A1,SLC25A19,SLC31A1,SLC39A10,SLC7A5,SMAD4,SMAD5,SMAD9,SMARCAL1,SMG1,SOD2,SOS1,SPOUT1,SPRY1,SPRY4,SQSTM1,SRC,SRGAP3,STIM2,STK3,SYNE2,TADA3,TAF6L,TARBP2,TCTN3,TEAD1,TEFM,TELO2,TERF1,TERF2,TFAM,TGFB1,TGM2,TMOD3,TP53,TP53RK,TPP2,TRPM4,TSPYL1,TWNK,TYMP,UHRF1,UIMC1,UNG,UTF1,UTRN,VAC14,VEZF1,VEZT,VPS26A,XPC,YAP1,ZC3HC1,ZNF148	CD73, CD118, CD221
Organismal death	6.93E-09	256	ABCB10,ABCC1,ABHD5,ABI1,ACADM,ACTL6A,ACTN4,ADGRG6,ADM,AFF4,AGL,AHI1,AKAP13,ALDH5A1,ARHGEF12,ARRB1,ATF2,ATF6,ATF6B,ATN1,ATP6V0C,ATP7A,B4GALT1,BARD1,BECN1,BIRC5,BIRC6,BRCA1,CASK,CASZ1,CAV1,CBS/CBSL,CC2D1A,CCDC134,CCNA2,CCNB2,CDK2,CDK2AP1,CEBPD,CHMP5,CIT,CLCN7,CLN3,CNOT3,CNTNAP1,COPS8,CPOX,CSK,CTNNA2,CUX1,DAB2,DAXX,DDR1,DIAPH3,DMD,DPAGT1,DVL2,ECD,ECSIT,EEF1E1,EFEMP1,EHD3,EHD4,EIF4E2,ELOVL1,ELOVL4,EP400,EPHB2,ERBIN,ERCC1,ERCC6,ERCC6L,ERF,ERRFI1,EXTL3,FITM2,FLVCR1,FNDC3B,FOS,FOSL1,FTL,GAS2L3,GATA3,GBE1,GCLM,GFAP,GJA1,GJC1,GLCE,GNG5,HEXB,HMGCL,HSBP1,HSD3B7,HSPG2,HYAL2,IGF1R,IL27RA,INO80,JUNB,KIF1A,KIF20A,KIF3A,KRT8,LAMC1,LIFR,LIMA1,LIMK1,LRP6,LRP8,LRRC8A,MADD,MAN2A1,MAP1B,MBNL1,MBTPS1,MCL1,MCM3AP,MED24,MELK,MKI67,MMACHC,MTA2,MTERF3,MTF2,MTHFD2,MYH10,MYH9,MYO18A,NCAPG2,NCAPH2,NCOR1,NDC80,NEBL,NF1,NF2,NFATC1,NFIX,NLE1,NOA1,NOP53,NOTCH2,NSUN4,NT5E,NUS1,OAT,PAFAH1B1,PAM,PANK1,PARL,PATZ1,PCYT1A,PDSS2,PEA15,PEX5,PFKM,PGP,PI4KA,PINX1,PISD,PITPNA,PKD2,PLCB1,PLCG1,PLIN2,PLXND1,PNPLA2,PNPLA8,POLB,POLG,POLR2A,POMGNT1,POMGNT2,PPP1R13L,PRKCD,PRMT1,PRPF31,PSMB4,PSMG1,PTBP2,PTK2,PTPN2,PTPN9,PXN,PYGO2,RAD54B,RAD54L,RAF1,RAI1,RB1,RBBP6,RBM38,RFK,RICTOR,RIF1,RPS6KB2,SCARB1,SCD,SHB,SLC16A2,SLC19A1,SLC19A3,SLC20A1,SLC25A19,SLC31A1,SLC39A10,SLC7A5,SMAD4,SMAD5,SMAD9,SMARCAL1,SMG1,SOD2,SOS1,SPOUT1,SPRY1,SPRY4,SQSTM1,SRC,SRGAP3,STIM2,STK3,SYNE2,TADA3,TAF6L,TARBP2,TCTN3,TEAD1,TEFM,TELO2,TERF1,TERF2,TFAM,TGFB1,TGM2,TMOD3,TP53,TP53RK,TPP2,TRPM4,TSPYL1,TWNK,TYMP,UHRF1,UIMC1,UNG,UTF1,UTRN,VAC14,VEZF1,VEZT,VPS26A,XPC,YAP1,ZC3HC1,ZNF148	CD73, CD118, CD221

© 2000–2020 QIAGEN. All rights reserved.

Supplementary Table 16A: Network analysis – 3D-cultured NCI-H23 cells. See Supplementary Table 16A

Supplementary Table 16B: Network analysis – 2D-cultured NCI-H23 cells. See Supplementary Table 16B
